# Accuracy of tropical peat and non-peat fire forecasts enhanced by simulating hydrology

**DOI:** 10.1038/s41598-022-27075-0

**Published:** 2023-01-12

**Authors:** Symon Mezbahuddin, Tadas Nikonovas, Allan Spessa, Robert F. Grant, Muhammad Ali Imron, Stefan H. Doerr, Gareth D. Clay

**Affiliations:** 1 Department of Renewable Resources, University of Alberta, Edmonton, AB T6G 2E3 Canada; 2 Department of Geography, Centre for Wildfire Research, Swansea University, Singleton Park, Swansea, SA2 8PP UK; 3 Faculty of Forestry, Universitas Gadjah Mada, Jalan Agro No. 1 Bulaksumur, Yogyakarta, 55281 Indonesia; 4 Department of Geography, School of Environment, Education and Development, University of Manchester, Oxford Road, Manchester, M13 9PL UK

**Keywords:** Hydrology, Ecological modelling, Fire ecology

## Abstract

Soil moisture deficits and water table dynamics are major biophysical controls on peat and non-peat fires in Indonesia. Development of modern fire forecasting models in Indonesia is hampered by the lack of scalable hydrologic datasets or scalable hydrology models that can inform the fire forecasting models on soil hydrologic behaviour. Existing fire forecasting models in Indonesia use weather data-derived fire probability indices, which often do not adequately proxy the sub-surface hydrologic dynamics. Here we demonstrate that soil moisture and water table dynamics can be simulated successfully across tropical peatlands and non-peatland areas by using a process-based eco-hydrology model (*ecosys*) and publicly available data for weather, soil, and management. Inclusion of these modelled water table depth and soil moisture contents significantly improves the accuracy of a neural network model in predicting active fires at two-weekly time scale. This constitutes an important step towards devising an operational fire early warning system for Indonesia.

## Introduction

Tropical peatland and forest fires in Indonesia are strongly associated with episodic drought events^1^. Indonesia has the largest coverage of tropical peatlands (206,950 km^2^)^2^ of which 10–15% are affected by fires each year^2–4^. Such fires can emit substantial amounts of greenhouse gases during drought years (up to about 5–10% of global emissions of CO_2_ from all human activities including agricultural land uses)^3–5^, cause significant increases in morbidities and mortalities through smoke haze pollution^6–8^ and degrade biodiversity^9,10^. These episodic events also have profound negative impacts on the economy^11^ and ecosystem services^9,10^ of the affected areas.

Water table depth (WTD) and peat moisture contents are predominant biophysical controls over the occurrence of peat and forest fires in tropical peatlands^12–14^. Prolonged droughts in Indonesia are caused by El-Niño and/or positive Indian Ocean Dipole (IOD) events which result in cooler-than-normal sea surface temperatures, and thus reduced precipitation^1,15,16^. Exacerbated by extensive peat drainage for agriculture and plantation establishment, these droughts can trigger severe peatland fires by lowering the WTD and hence desiccating surface and sub-surface peats^17,18^. Loss of fire-resistant natural forest canopy cover through large-scale deforestation can also alter the micro-meteorology and hydrology of tropical peatlands, further aggravating the fire situation^19^. The severe drought episodes of 2015 and 2019 across Indonesia, for example, caused by strong El-Niño events and positive IOD, led to a major and damaging increase in peatland fires, highlighting an urgent need to develop operational systems to forecast potentially severe fire events to mitigate the impacts of fire and haze^20,21^.

Despite the recognized importance of peatland hydrology in determining fire activity in Indonesia^13,14,22–24^, there has been little progress to date in developing an operational fire early warning system for Indonesian peatlands that takes advantage of recent advances in soil hydrology modelling^14,25–27^. Although seasonal variations in near-surface peat moisture contents, and rates of groundwater recharge have been shown to be correlated with fire occurrence patterns in tropical peatlands^14,22,28,29^, the explicit inclusion of hydrologic dynamics into peat fire forecasting models remains constrained by several factors. These factors include: a scarcity of available (i) measurements of seasonal variations in WTD and peat moisture contents, (ii) measurements of peat soil physical and hydrological properties such as bulk density, organic carbon, moisture retention, and hydraulic conductivity, and (iii) process-based peat hydrology models that can predict hydrologic dynamics across temporal and spatial scales^22,28^.

**Figure 1:**
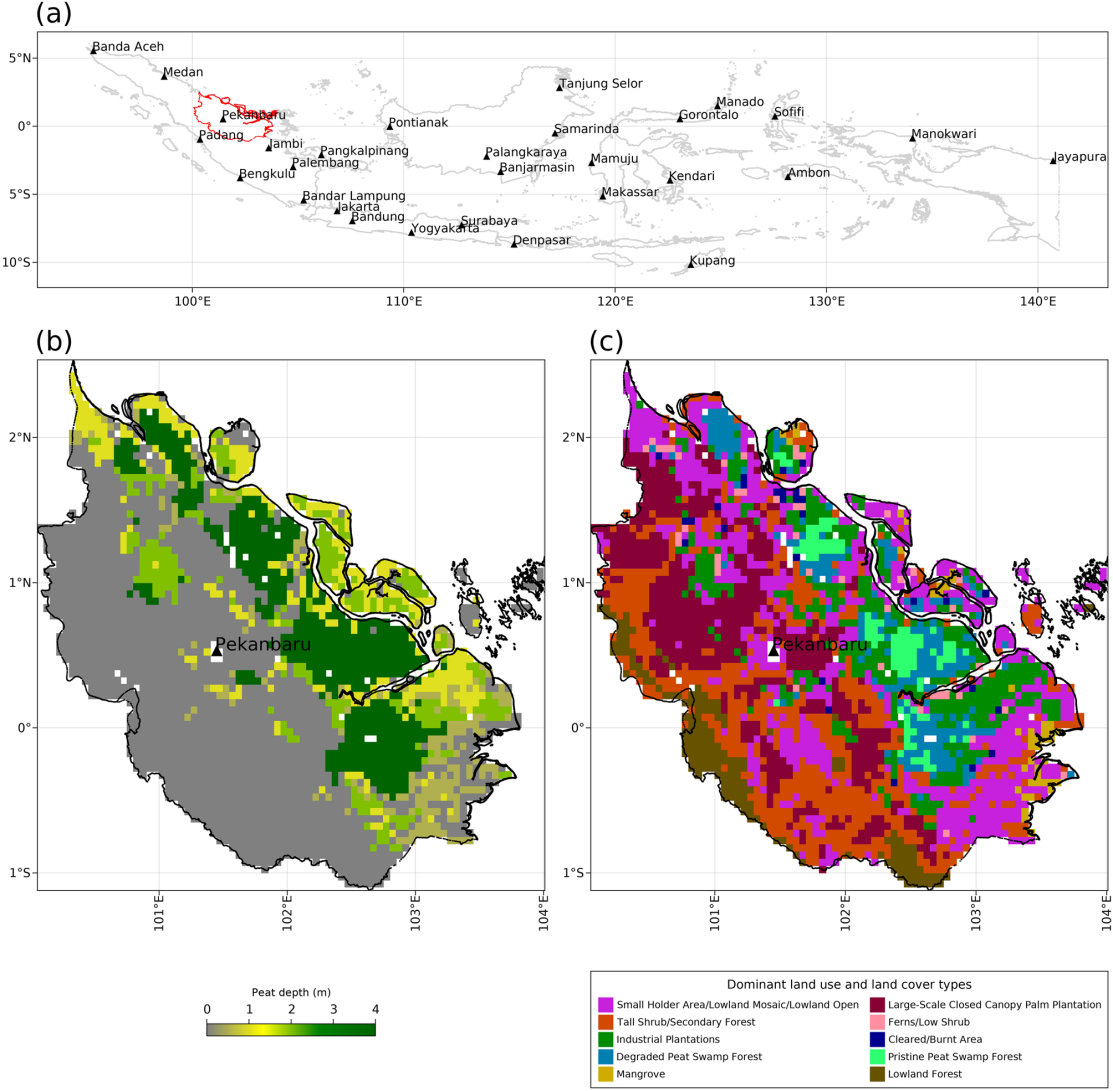
Study area for modelling tropical peatland hydrology. (**a**) Location (red boundary) with major city names (labelled black triangles) (**b**) minimum peat depth, and (**c**) dominant land use and land cover types of Riau province, Sumatra, Indonesia. The maps in (**a**–**c**) were created by using Julia Programming Language version 1.6.7 (https://julialang.org/downloads/).

In this study, we explore whether simulated peatland hydrology can be used to improve fire activity predictions in tropical regions. Using machine learning, we predicted daily fire activity at a resolution of 0.05° (ca. 5 kms) for the tropical peatlands of Riau province (east-central Sumatra, Indonesia) (Fig. 1) as a function of (i) weather variables only versus (ii) the same weather variables, plus specific outputs from an established process-based tropical peatland hydrology model called *ecosys*^26,27^ viz. soil moisture and WTD (Supplementary Figs. S1, S2). The *ecosys* model was parameterised and benchmarked from available scientific data, which although not entirely perfect in terms of coverage and quality, nonetheless allowed us to gain valuable insights into the strengths, limitations, and scalability of *ecosys*. Our analyses demonstrate that the inclusion of *ecosys* hydrologic dynamics significantly enhances the prediction of active fires in Riau, compared with using weather variables only, especially during severe drought.

## Results and discussion

### Modelling hydrologic dynamics

**Figure 2:**
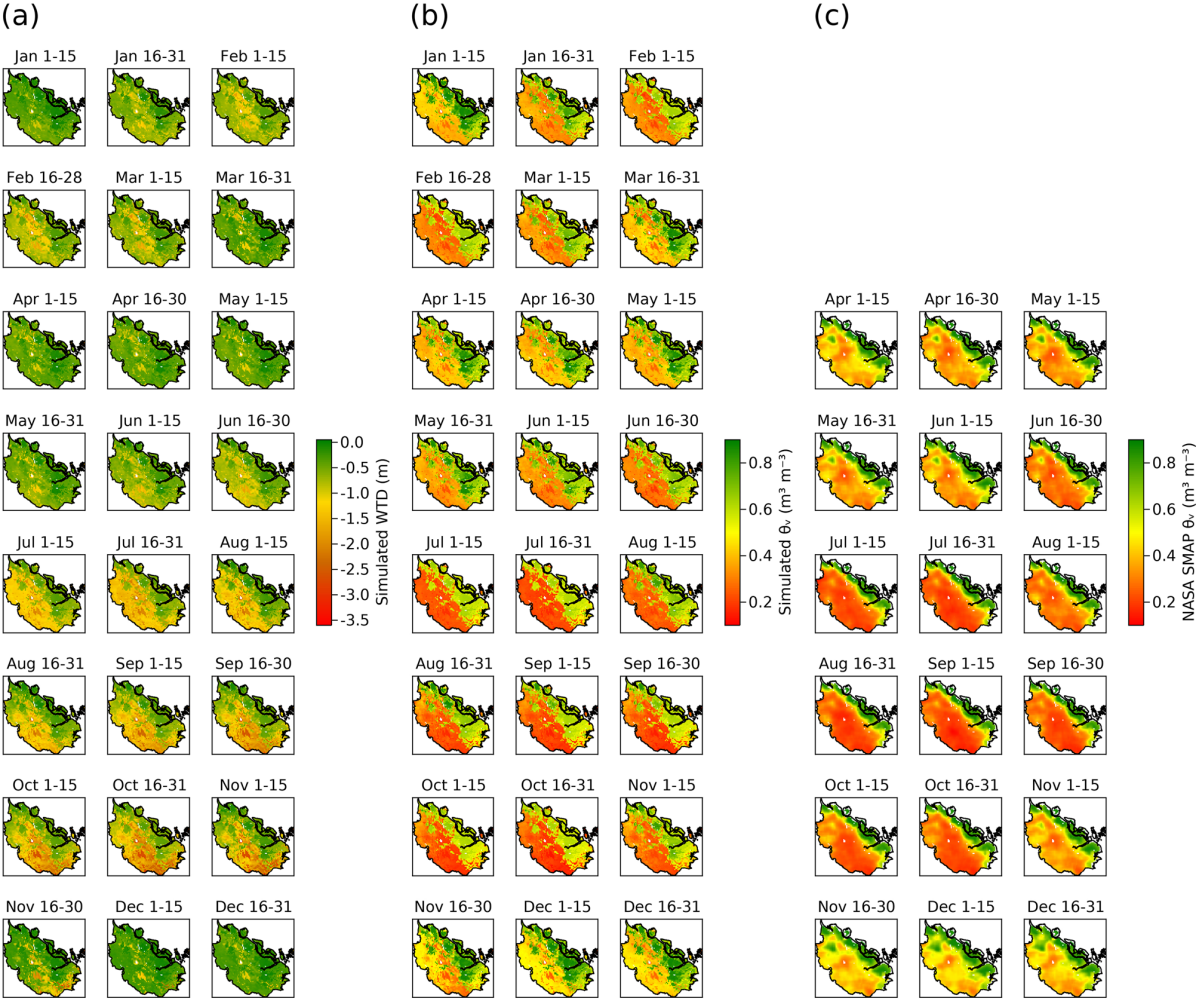
Spatial distribution of seasonal variations in tropical peatland and non-peatland water table depth and soil moisture contents. Half-monthly averaged (**a**) simulated water table depth (WTD) (**b**) simulated near-surface (0–0.05 m depth) soil moisture content (θ_v_), (**c**) National Aeronautics and Space Administration (NASA) Soil Moisture Active Passive (SMAP) near-surface soil moisture contents during 2015 across Riau province, Sumatra, Indonesia. Negative water table depths are depths below the ground surface. The maps in (**a**–**c**) were created by using Julia Programming Language version 1.6.7 (https://julialang.org/downloads/).

**Figure 3:**
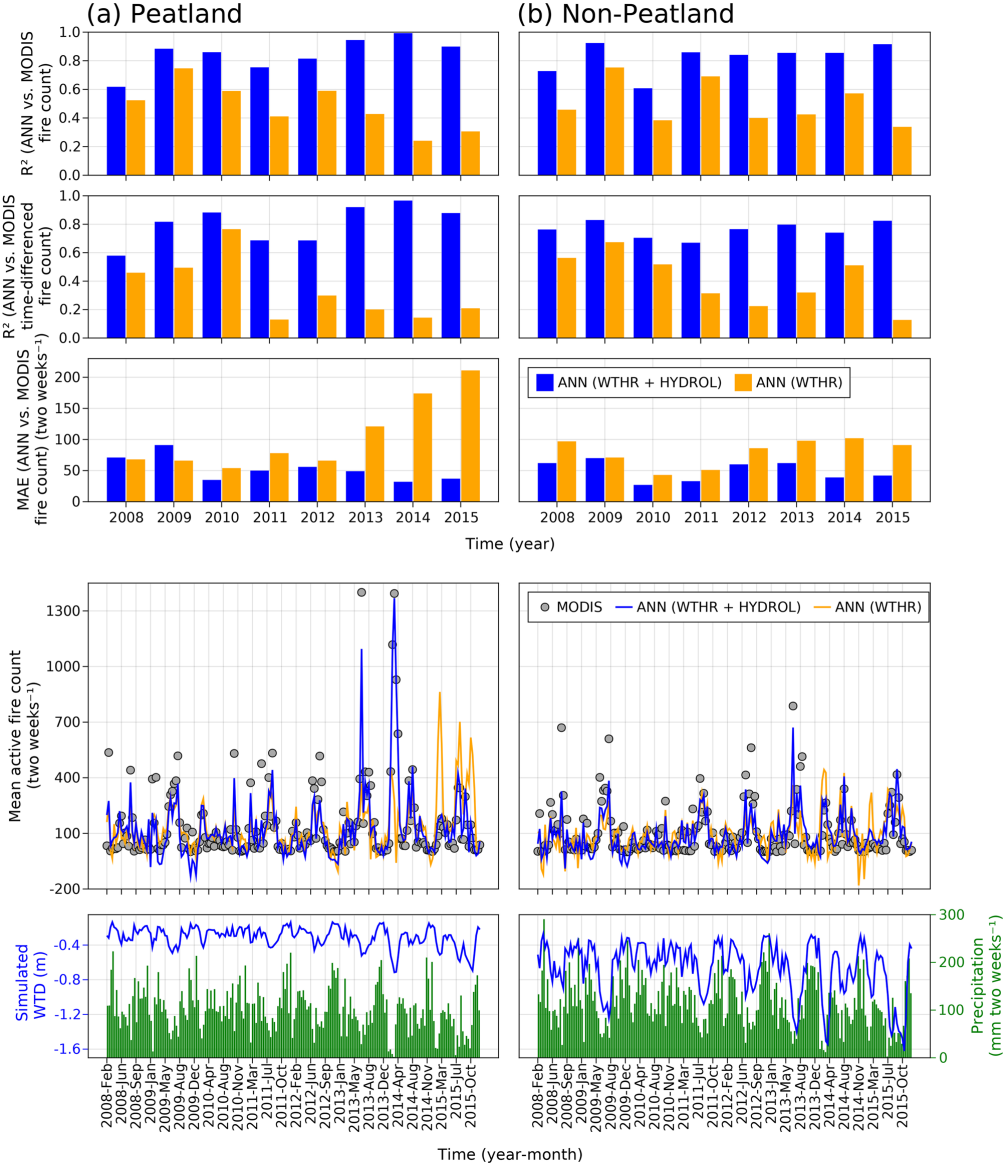
Performance of tropical peatland and non-peatland fire prediction models with and without the inclusion of hydrology. Coefficients of determination (R^2^) for real-time and time-differenced (*n*_t+1_ − *n*_t_; where, *n* = fire count and t = two-week time-step) predicted vs Moderate Resolution Imaging Spectroradiometer (MODIS) active fire count and mean absolute errors for predicted vs MODIS active fire count averaged at two-week time step and accumulated over (**a**) peatland and (**b**) non-peatland grids. MODIS, and artificial neural network (ANN) predicted active fire count averaged at two-week time steps, precipitation summed over two-week time steps, and simulated water table depth (WTD) averaged at two-week time steps over (**a**) peatland and (**b**) non-peatland grids during 2008–2015 across Riau province, Sumatra, Indonesia. ANN (WTHR + HYDROL) = ANN model featured by both weather and hydrology variables, ANN (WTHR) = ANN model featured by only weather variables. Negative water table depths are depths below the ground surface.

The *ecosys* model simulated WTD drawdown and desiccation of near-surface (0–0.05 m depth) peat and mineral soil at a 0.05° resolution across Riau during both the short and the long dry seasons in 2015 (Fig. 2). A soil layer with organic matter content > 65% was defined as a peat soil layer^45^ and a layer with a lower organic matter content was defined as a mineral soil. All grid-cells in which soil organic matter contents in all soil layers from surface down to 0.3 m or deeper exceeded 65% were defined as peatland grids^45^ and the remaining grid-cells were defined as non-peatland grids. 2015 was a drought year with a short dry season between mid-January and late-February and a prolonged and intense dry season that lasted from mid-June to late October with a brief rainy period in early August (Supplementary Fig. S3). Beside seasonal variability, modelled WTD was generally shallower and near surface soil moisture content was higher in peatland grid cells when compared to the non-peatland grid cells (Fig. 2). The near-surface peat and mineral soil moisture contents sensed by National Aeronautics and Space Administration (NASA) Soil Moisture Active Passive (SMAP) also showed similar seasonal and spatial variations across Riau during 2015 (Fig. 2). The seasonal variation in simulated near-surface peat and mineral soil moisture contents was strongly correlated with SMAP peat and mineral soil moisture in most of the grids as indicated by a median correlation coefficient of 0.7 between daily modelled and SMAP soil moisture contents across Riau (Fig. 2) (Supplementary Fig. S4). However, modelled daily mineral soil moisture contents across non-peatland grids showed stronger correlations with SMAP than did modelled daily moisture contents in peatland grid cells i.e., median correlation coefficient between daily modelled and SMAP soil moisture contents was 0.56 for peatland grid cells vs. 0.79 for non-peatland grid cells across Riau (Fig. 2) (Supplementary Fig. S4). Near zero or even negative correlations in modelled vs. SMAP peat moisture contents occurred in about 25% of the peatland grids along the eastern coast of the province (Fig. 2) (Supplementary Fig. S4). Like modelled soil moisture contents, SMAP soil moisture contents were also generally higher in peatland than in non-peatland grid-cells (Fig. 3). Seasonal variations in WTD across both peatland and non-peatland grids followed the same trends in modelled and SMAP soil moisture contents (Fig. 3). Average modelled WTD for grid-cells under pristine and degraded peat swamp forests was 0.21 m below the ground which was shallower than average modelled WTD under industrial plantations (0.47 m below the ground) and small holders’ farmlands (0.56 m below the ground) (Figs. 1, 2).

Seasonal variations in WTD and near-surface soil moisture contents were modelled by solving the balance between vertical water exchange (precipitation vs. evapotranspiration) and lateral water exchange (lateral recharge vs. discharge) (Supplementary Figs. S1–S6). During the rainy season, precipitation exceeded simulated evapotranspiration that caused net vertical water recharge, which raised modelled WTD and drove lateral discharge from the modelled peat and mineral soil profiles^26^. The rate of lateral discharge was controlled by moisture holding capacities and hydraulic conductivities of different vertical modelled soil layers and the hydraulic gradient between WTD within the modelled peat and mineral soil profiles and an external boundary WTD termed as WTDx^26^. The WTDx was a pre-defined lateral boundary condition, which mimicked the WTD of an adjacent watershed or a canal with reference to the surface of a modelled peat or mineral soil profile^13,26^ (Supplementary Table S1). Modelled WTD was the depth within a modelled peat or mineral soil profile below which the modelled soil layers were completely saturated. With the onset of the dry season, lateral discharge exceeded the vertical recharge (precipitation minus evapotranspiration), which caused a drawdown of modelled WTD^26^. Deepening of modelled WTD reduced the hydraulic gradient between WTD and WTDx, which gradually slowed down lateral discharge towards the end of the dry season^17,18^.

Tropical peatland WTD dynamics typically follows seasonal variation in near-surface peat moisture contents^29^. Since variations in simulated peatland WTD followed similar trends in both simulated and SMAP near-surface peat moisture contents, it could be a proxy validation of adequate simulation of WTD dynamics across peatlands in Riau. Furthermore, the large-scale hydrology simulation across Riau was built upon establishing the accuracy of *ecosys* model in simulating hydrologic dynamics over contrasting tropical peatland sites in this study (Supplementary Figs. S6 and S7), which also supports previous tropical peatland hydrology benchmarks of *ecoys*^26,27^. Deeper WTD in industrial plantations and smallholders’ farmlands were modelled by simulating effects of artificial drainage by setting up deeper WTDx as lateral boundary conditions (Supplementary Table S1)^26^. Long-term site measurements across peatlands in Sumatra showed similarly deeper WTD due to artificial drainage across industrial plantations and small holders’ farmlands when compared with peat swamp forests^30–34^ (Supplementary Fig. S7). Deeper WTD in non-peatland grid-cells when compared with peatland grids was also simulated by setting up deeper WTDx, so that deeper lateral drainage was allowed in non-peatland than in peatland grid-cells^26,35^.

Higher near-surface soil moisture contents in peatland than in non-peatland grids were modelled by simulating differential water holding capacities between peat and mineral soils from model inputs of soil hydrologic properties into basic soil physics algorithms of water retention and movement through porous media (Fig. 2) (Supplementary Fig. S1) (Supplementary Eqs. 1–23)^26^. Divergence between simulated and SMAP soil moisture contents in the coastal peatland grids was exacerbated by desiccation of simulated near-surface peat during the two dry seasons of 2015 as opposed to minimal or no near-surface peat desiccation in SMAP data (Fig. 2) (Supplementary Fig. S4). For those coastal peatland grids to remain saturated or close to saturation, there had to be significant moisture gains during the dry season through lateral recharge either from the coastline or from upland non-peatland areas. This anomaly may be explained by the fact that *ecosys* grid-cells are stand-alone, that is, they are not connected through either lateral surface or sub-surface water exchanges, thereby potentially preventing accurate simulations of lateral moisture gains from upland areas. Grid cells can be interconnected to form complex landscapes in *ecosys* simulations, which was not computationally feasible at the scale of this study. However, tropical peatlands in Riau like much of Indonesia are ombrotrophic (rain-fed) and are not known to receive significant moisture through lateral recharge^2,31,36^. Furthermore, during the dry season in 2015, the upland non-peatland areas also had drier soils that meant no potential lateral run-off or discharge from those areas (Fig. 2) (Supplementary Fig. S4). Additionally, most of these grid-cells were too far (> 10 km away) from the coast to have significant moisture gains through lateral recharge from the coastline (Fig. 2) (Supplementary Fig. S4).

An alternative explanation for continuous near-saturation of near-surface peat throughout the year across those coastal grid-cells could be due to high moisture retention capacities and very low saturated hydraulic conductivities that prevented pore drainage from those SMAP soil layers. However, near-surface peats in tropical peatlands are porous with low moisture retention capacities and high saturated hydraulic conductivites^22,36^, which would, in turn, lead to *ecosys* simulating rapid pore drainage and thereby preventing near-saturation moisture retention during dry seasons. Furthermore, the SMAP soil moisture contents across Riau have yet to be validated against field measurements^29^. Overall, the two streams of uncertainty ‒ one stemming from the stand-alone grid structure of the *ecosys* model selected for this study, and the other from the lack of benchmarked SMAP data—made it difficult to assess the validity of SMAP sensed high moisture contents in coastal grid-cells during the dry seasons of 2015.

### Integrating hydrologic dynamics into fire prediction

We compared two artificial neural network (ANN) models^37^, ANN (WTHR) and ANN (WTHR + HYDROL), to examine whether and how hydrologic dynamics would improve the predictive capability of fire forecasting over tropical peatlands (Supplementary Fig. S2). The ANN (WTHR) model used only weather variables, while the ANN (WTHR + HYDROL) model used both weather and hydrology variables as predictors (see Methods for details) (Supplementary Fig. S2). Each model was trained to predict active fire counts as detected by Moderate Resolution Imaging Spectroradiometer (MODIS) at a two-week time step for each modelled grid-cell from 2008 to 2015. Predicted and validation data were summed up over all the peatland and non-peatland grids for comparisons between the two parallel fire models in their capacity of predicting MODIS active fire count at two-week time steps.

The ANN (WTHR + HYDROL) performed consistently better than ANN (WTHR) in predicting MODIS active fire counts over a range of weather and fire years from 2008 to 2015, as indicated by higher coefficient of determination (*R*^2^) (*R*^2^ = ~ 0.8 for ANN (WTHR + HYDROL) vs. *R*^2^ = ~ 0.4 for ANN (WTHR)) and lower mean absolute errors (MAE) (MAE = ~ 40 two-weeks^−1^ for ANN (WTHR + HYDROL) vs. MAE = ~ 100 two-weeks^−1^ for ANN (WTHR)) for ANN (WTHR + HYDROL) predicted vs. validation fire counts (Fig. 3)^14^. ANN (WTHR) model was off by at least two weeks in predicting MODIS active fire count in most years as indicated by smaller *R*^2^ for ANN (WTHR) vs. predicted time-differenced (*n*_t+1_ − *n*_t_; where, *n* = fire count and t = two-week time-step) fire counts (Fig. 3). Fire occurred in almost every dry season across peatlands in Riau between 2008 and 2015 (Fig. 3)^7^. When simulated WTD was close to or deeper than 0.4 m below the ground, there was a fire event (Fig. 3), which confirmed the significance of previously reported WTD thresholds for fire occurrence^12^. During those years, Riau had two distinct fire seasons—a short one in February–March and a long one between June and September (Fig. 3)^15^. The two most extreme but very episodic peatland fire events within those years occurred during June-July in 2013 and February–March in 2014 (Fig. 3)^7^. ANN (WTHR + HYDROL) model was significantly more skillful than ANN (WTHR) in predicting those two extreme episodic fire events (Fig. 3). Unlike those extreme fire events in non-drought years, ANN (WTHR) model overestimated peatland fire counts during the drought year of 2015 (Fig. 3).

**Figure 4:**
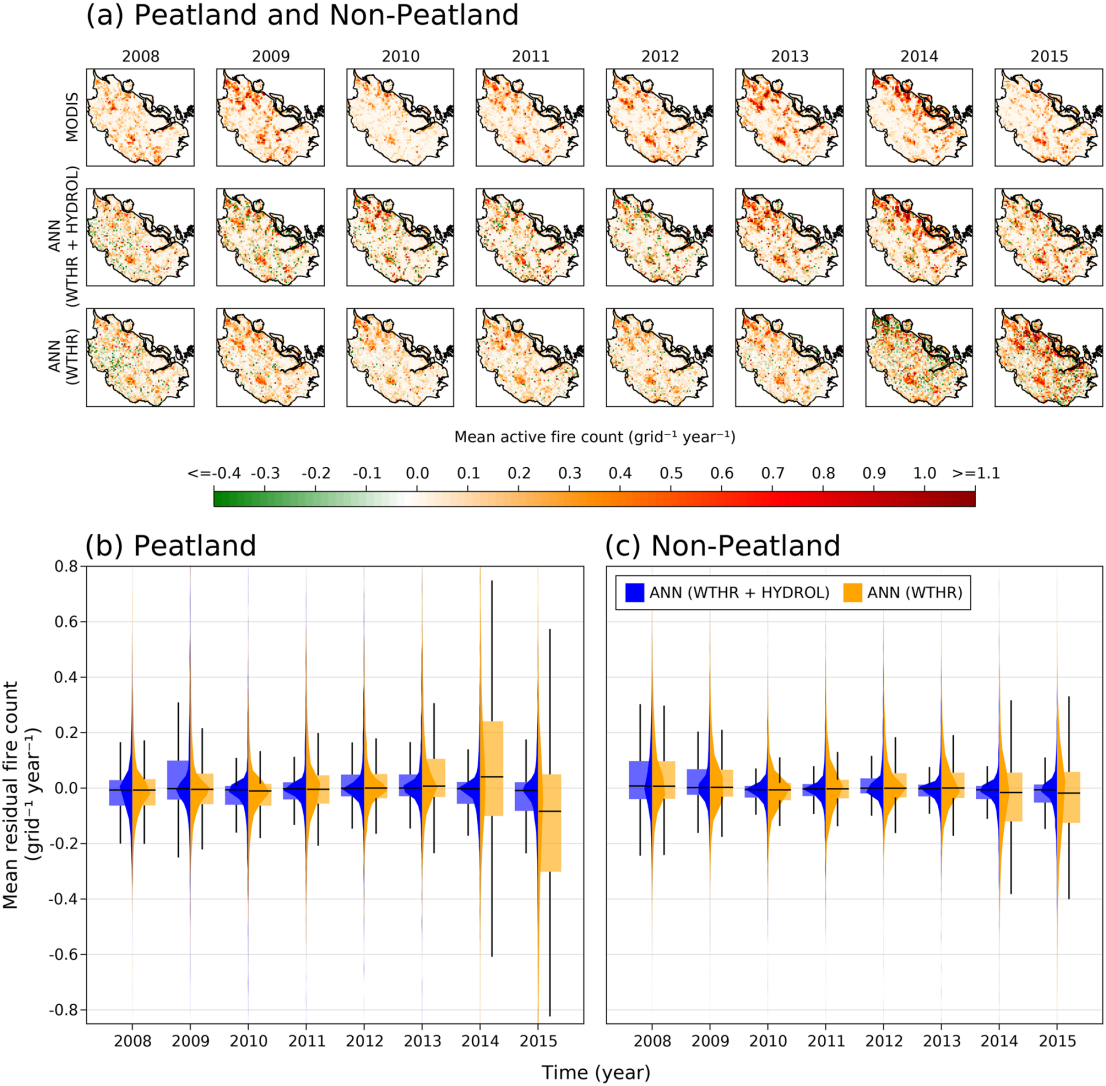
Spatial distribution of active tropical peatland and non-peatland fires. (**a**) Mean annual Moderate Resolution Imaging Spectroradiometer (MODIS), and artificial neural network (ANN) predicted active fire count during 2008–2015 for each grid across Riau province, Sumatra, Indonesia. Distribution of mean annual residual fire count (= MODIS—predicted) over (**b**) peatland and (**c**) non-peatland grids as depicted in vertical probability distribution plots and paired box and whisker plots. ANN (WTHR + HYDROL) = ANN model featured by both weather and hydrology variables, ANN (WTHR) = ANN model featured by only weather variables. The maps in (**a**) were created by using Julia Programming Language version 1.6.7 (https://julialang.org/downloads/).

The spatial distribution of mean annual MODIS versus predicted fire counts further demonstrated that ANN (WTHR) model missed most of the peatland fire hotspots during 2013 and 2014 and rang false alarms during 2015 (Fig. 4). The probability distribution curves showed wider distribution of residuals between MODIS and ANN (WTHR) predicted fire counts, further indicating larger and widespread errors in fire prediction when hydrology variables were not included (Fig. 4). Medians of mean annual residuals for ANN (WTHR) model were higher in 2013 and 2014 (indicating under prediction) and lower in 2015 (indicating over prediction) as compared to ANN (WTHR + HYDROL), more so in peatland than in non-peatland grids (Fig. 4). Further to peatlands, inclusion of hydrologic dynamics did also significantly and consistently improve the accuracy of fire count prediction over non-peatland grids in Riau during 2008–2015 (Figs. 3, 4).

Our results clearly demonstrated the direct inclusion of seasonal and interannual variations in WTD and near-surface soil moisture contents significantly improved the predictive skill of a fire activity forecast model based on weather variables alone across the tropical peatlands and forests of Riau in both drought and non-drought years (Figs. 3, 4). Improved prediction of fire activity in drought years is important as these years typically produce the most fires and experience the worst impacts from fires. The improved short-term prediction of peatland fire events in 2013 and 2014 which occurred during non-drought years^7^ is also important, as such events are generally more difficult to predict^6^.

Mechanistically, the inclusion of WTD and near-surface soil moisture significantly improve fire forecasting power in tropical non-peatlands because the hydrologic dynamics provide the fire model with explicit information on surface litter dryness and hence flammability, which weather variables cannot directly capture. Regarding tropical peatlands, we note that the improved performance of the fire model with peat hydrology included reflects two issues. Firstly, seasonal variations in WTD and near-surface soil moisture contents of peats are more gradual and hence often lag variations in precipitation and surface evaporation driven by temperature changes, and these responses depend upon the balance between vertical and lateral water fluxes in peats. Secondly, variability in daily tropical peatland WTD and soil moisture contents are not only controlled by the weather variables, but also by hydrologic properties of soil and both natural and artificial drainage.

Our study demonstrates that using publicly available data, a scalable and satisfactorily validated process-based soil hydrology model such as *ecosys* can be used to predict hydrologic dynamics and hence fire patterns in tropical peatlands and forests in Indonesia. Despite (i) the dearth of soil, weather and land use data for initializing and testing the model, and (ii) the simplified selection of user defined lateral model boundary conditions to optimize computational requirements (i.e. no lateral water exchanges between adjacent grid-cells), which introduced potential uncertainties into the *ecosys* simulations, we demonstrate here that outputs from a process-based soil hydrology model significantly increases the skill of fire forecasts in tropical ecosystems. The methodology presented here can easily be scaled up to provide the framework for skillful early fire warning system for the whole of Indonesia and other tropical countries exhibiting a complex land-cover mix of peatlands, non-peatlands, degraded and pristine forests. For instance, *ecosys* can be driven by seasonal forecasts such as those available through the Copernicus Data Server (CDS) (https://cds.climate.copernicus.eu/cdsapp#!/dataset/seasonal-original-single-levels?tab=form) to forecast seasonal variations in hydrology, which would then feed into the fire forecasting model. Beyond skillful fire prediction, an *ecosys*-based fire early warning system could also be used to assess the impact of different management interventions to mitigate against potentially severe droughts in future such as reforesting deforested peatlands and rewetting drained peat swamps.

## Methods

### Large-scale hydrology modelling

Seasonal variations in WTD and near-surface peat and mineral soil water contents were simulated by using the process-based ecosystem model *ecosys* that has previously been validated across tropical^26,27^, temperate^38^ and boreal^39–41^ peatlands (see Supplementary Materials S1 for more details). The *ecosys* model was scaled to 0.05° × 0.05° grids across the Riau province following site-level validation and benchmarking of *ecosys* across contrasting tropical peatland sites (see Supplementary Materials S1 for details on site-level validation)^26,27^.

The model was driven at each grid-cell by the following inputs: landform, soil, weather and dominant land use and land cover type. These data were obtained from published literature and datasets to represent relevant field conditions (Supplementary Figs. S2, S3, S9–S18) (Supplementary Table S2). Model inputs for dry bulk density, and soil organic carbon for the 15 vertical layers in each peatland grid were derived from a comprehensive literature review of peat physical properties observed at different sites and depths across Indonesian peatlands under different land use, land cover and land management (Supplementary Figs. S9, S10) (Supplementary Table S3). Model inputs for non-peatland mineral soil properties were derived from publicly available SoilGrids250m dataset^42^ (Supplementary Figs. S10–S13). Model inputs for soil moisture retention parameters for each layer of the peatland grids were estimated through parameter optimization for each grid-cell at each vertical layer (see Supplementary Materials S1 for details) (Supplementary Figs. S14–S17). The moisture retention model parameters for mineral soil layers were estimated using the same procedure as in peatland soils but using different sets of moisture retention data from pedo-transfer functions as reported for tropical mineral soils^43^ (Supplementary Figs. S14–S17).

Model inputs for saturated hydraulic conductivities for all vertical soil layers in both peatland and non-peatland grids were derived using pedo-transfer functions (see Supplementary Materials S1 for details) (Supplementary Fig. S18). Lateral saturated hydraulic conductivity of a layer of a grid soil profile was assumed identical to the vertical saturated hydraulic conductivity for that layer of that grid profile. Each of the modelled grid-cells was stand-alone and did not exchange moisture between two adjacent grid-cells. The lateral surface run-off from each grid was driven by surface geometry, slope, and depth of ponded water (see Supplementary Materials S1 for details). The rate of subsurface lateral discharge from each grid-cell was driven by hydraulic gradient between modelled WTD and the lateral boundary water table, WTDx; and the saturated hydraulic conductivity of each layer through which lateral discharge occurred. For peatland grids, model inputs for the lateral boundary conditions of WTDx were estimated based on available average long-term WTD measurements during dry seasons across tropical peatlands in Sumatra^30–34^ (Supplementary Table S1). WTDx for all non-peatland grids except those under mangroves was set at 6 m depth to allow lateral discharge along the whole vertical mineral soil profiles (Supplementary Table S1).

Each grid-cell was initialised with a dominant plant functional type (PFT) throughout each model run reflecting phenology, plant physiology, land use and land cover types and common agricultural and forest management practices across Riau (Supplementary Table S4). Each model run was preceded by 56-year spin-up cycle using a repeated weather sequence from ERA5 global reanalysis meteorological observations and Climate Hazards group Infrared Precipitation with Stations (CHIRPS)^44^ precipitation data, which then continued to the production runs for the simulation years (2008–2015) (Supplementary Table S2). Modelled outputs for daily near-surface soil moisture contents during April-December of the drought year 2015 were averaged for the modelled surface litter layer (thickness = 0.02 m) and the top two modelled soil layers (thickness = 0.03 m) below the surface litter layer. The average daily modelled peat or mineral soil moisture contents in each grid-cell were compared against available daily near-surface (0–0.05 m depth) peat or mineral soil moisture contents from SMAP dataset corresponding to the same grid-cell. Model accuracy was assessed by correlation analyses between daily modelled and SMAP near-surface soil moisture contents.

### Fire forecasting with and without hydrology

We built two parallel artificial neural network (ANN) models, with and without including simulated WTD and soil moisture as predictors, to predict MODIS active fire count from 2008 to 2015 for each modelled grid-cell across Riau at a two-week time step. The ANN (WTHR) model used weather variables only, e.g., average air temperature, radiation, wind speed and relative humidity, and total precipitation over each of the two-week time steps, as features or predictor variables (Supplementary Fig. S2). Precipitation summed over four weeks preceding each model time step was also used as a feature in ANN (WTHR) model to represent any pre-drying condition. The ANN (WTHR + HYDROL) model used two additional features, e.g., simulated near-surface (0–5 cm depth) soil moisture contents and WTD averaged over each two-week time step, along with the weather variables in ANN (WTHR) (Supplementary Fig. S2).

The two ANN models were trained for each grid-cell using leave-one-year-out strategy. Each of the fully connected ANN regression models comprised of four layers—an input and an output layers and two hidden layers in between^37,46^. Features for each of the ANN (WTHR) and ANN (WTHR + HYDROL) models were represented by 6 and 8 nodes respectively^37,46^. The two hidden layers in both the models had 32 and 16 neurons respectively. The input layer and the two hidden layers of each ANN model had an activation function ‘relu’^37^. The loss function for each ANN model was mean squared error, which was optimised by using an optimizer ‘ADAM’^37,46^ with a learning rate of 0.001. Each of the two parallel ANN models was trained and validated by using leave-one-year-out strategy, where MODIS fire count data for one year was held out as validation dataset in each iteration. Early stopping strategy was used to avoid overfitting and to increase the generalization power of the model^37,46^.

Comparative model performance was evaluated by comparing out-of-sample coefficient of determination (*R*^*2*^) and mean absolute error (MAE). For this purpose, predicted and MODIS active fire counts at each two-week time step were pooled separately for all peatland grids and all non-peatland grids for each validation year between 2008 and 2018. The ANN (WTHR) and ANN (WTHR + HYDROL) models were then compared against each other using the *R*^2^ and MAE statistics generated while regressing accumulated modelled active fire counts vs. MODIS active fire count for each test year, separately for both peatland and non-peatland grids. To assess their temporal accuracies, the two ANN models were assessed by comparing predicted versus MODIS time-differenced (*n*_t+1_ − *n*_t_; where, *n* = fire count and t = two-week time step) fire counts.

## Supplementary Information

Supplementary Information.

## Data Availability

All model inputs and validation data are publicly available and can be downloaded from data sources (web links and/or published paper) cited within the main manuscript and the supplementary materials.

## Code availability

*Ecosys* source codes can be found at https://github.com/jinyun1tang/ECOSYS. Fire forecasting model codes can be found at https://github.com/4SAnalyticsnModelling/TOFEWSI-ECOSYS-ANN.
