# Supplementary material for: Accuracy of tropical peat and non-peat fire forecasts enhanced by simulating hydrology
Source: Sci Rep. 2023 Jan 12;13:619. doi: 10.1038/s41598-022-27075-0 (PMC9837124; doi:10.1038/s41598-022-27075-0)
Supplement: Supplementary file 1 — Supplementary Information. [file 41598_2022_27075_MOESM1_ESM.docx]

**Accuracy of tropical peat and non-peat fire forecasts enhanced by simulating hydrology**

**(Supplementary Materials)**

Symon Mezbahuddin^1^, Tadas Nikonovas^2^, Allan Spessa^2^, Robert F. Grant^1^, Muhammad Ali Imron^3^, Stefan H. Doerr^2^ & Gareth D. Clay^4^

*^1^Department of Renewable Resources, University of Alberta, Edmonton, AB, T6G 2E3, Canada*

*^2^Centre for Wildfire Research, Department of Geography, Swansea University, Singleton Park, Swansea, SA2 8PP, UK*

*^3^Faculty of Forestry, Universitas Gadjah Mada, Jalan Agro No. 1 Bulaksumur Yogyakarta 55281, Indonesia.*

*^4^Department of Geography, School of Environment, Education and Development, University of Manchester, Oxford Road, Manchester, M13 9PL, UK*

**1. Supplementary Methods**

**1. 1. Model description**

Seasonal variation in tropical peatland and non-peatland WTD and soil moisture contents in this study was simulated by using a process-based ecosystem model *ecosys* that was rigorously validated for its predictive capacity in simulating hydrologic dynamics across tropical^1,2^, temperate^3^ and boreal^4,5,6^ peatlands (Supplementary Fig. 1). The WTD in *ecosys* is the depth with respect to the modelled surface below which the modelled peat or mineral soil column is saturated. At this depth lateral water fluxes in *ecosys* are at equilibrium with the difference between the vertical influxes (precipitation) and effluxes (evapotranspiration) (Supplementary Fig. 1). Surface runoff in *ecosys* is modeled from surface water velocity, which is calculated from surface geometry, slope, and depth of ponded water that is calculated from surface water balance. Lateral discharge/recharge from and into the modelled peat or mineral soil profile is governed by hydraulic gradient between modelled WTD and an external WTD (WTDx) specified as a lateral boundary condition (Supplementary Fig. 1). The rates of these lateral water fluxes are also controlled by the hydraulic conductivity of the soil layer in which these fluxes occur.

Vertical surface boundary conditions in *ecosys* are defined by model inputs of weather variables, which includes precipitation, incoming solar radiation, air temperature, humidity, and wind speed. These variables drive energy balance calculations to give rise to vertical water effluxes of evapotranspiration from canopy, peat or mineral soil surfaces and surface litters (Supplementary Fig. 1). Vertical water effluxes in *ecosys* are also coupled with subsurface water fluxes through modelled root and peat or mineral soil profiles in a peat or mineral soil-plant-atmosphere water continuum^1,2,4^. Subsurface water fluxes through modelled peat or mineral soil profiles are driven by hydraulic gradients that arise from differences in peat or mineral soil water potentials at different points within the modelled peat or mineral soil profiles^1,4^. The rates of subsurface water fluxes through modelled peat or mineral soil profiles are also controlled by unsaturated hydraulic conductivities that are affected by peat or mineral soil moisture retention^1,4^. Peat or mineral soil moisture retention and unsaturated hydraulic conductivities in *ecosys* are simulated by using Mualem-van Genuchten model (Supplementary Equations 1-9)^4^. Parameters for the Mualem-van Genuchten model are obtained internally by optimizing model inputs for peat or mineral soil moisture retention properties^4^. The seasonal variation in WTD in *ecosys* is thus not prescribed, but prognostically simulated from the balance between vertical and lateral water fluxes at each model time step (hourly).

**Van Genuchten model for soil and peat moisture retention**^7^

$\theta_{v}=\theta_{r}+\left( \theta_{s}-\theta_{r} \right)\left[ 1+\left( \alpha\psi\right)^{n} \right]^{-m}$ **[Supplementary Equation 1]**

$S_{e}=\frac{\theta_{v}-\theta_{r}}{\theta_{s}-\theta_{r}}=\left[ 1+\left( \alpha\psi\right)^{n} \right]^{-m}$ **[Supplementary Equation 2]**

$\psi=\frac{\left[ S_{e}^{\frac{-1}{m}}-1 \right]^{\frac{1}{n}}}{\alpha}$ **[Supplementary Equation 3]**

**Mualem-van Genuchten model for soil and peat unsaturated hydraulic conductivity**^7^

$K_{r}=\frac{K_{unsat}}{K_{s}}$ **[Supplementary Equation 4]**

$K_{r}=S_{e}^{0.5}\left[ 1-\left( 1-S_{e}^{\frac{1}{m}} \right)^{m} \right]^{2}$ **[Supplementary Equation 5]**

**Ippisch modification of Mualem-van Genuchten model for soil and peat unsaturated hydraulic conductivity**^8^

$S_{c}=\left[ 1+\left( \alpha\psi_{e} \right)^{n} \right]^{-m}$ **[Supplementary Equation 6]**

$S_{e}=\frac{\left[ 1+\left( \alpha\psi\right)^{n} \right]^{-m}}{S_{c}};when\psi<\psi_{e}$ **[Supplementary Equation 7]**

$S_{e}=1;when\psi\geq\psi_{e}$ **[Supplementary Equation 8]**

$K_{r}=S_{e}^{0.5}\left[ \frac{1-\left( 1-\left( S_{c}S_{e} \right)^{\frac{1}{m}} \right)^{m}}{1-\left( 1-{S_{c}}^{\frac{1}{m}} \right)^{m}} \right]^{2}$ **[Supplementary Equation 9]**

**Pedo-transfer functions**

**For peat soils**

PEATCLASS = [1, 2, 3, 4, 5, 6, 7, 8, 9, 10] **[Supplementary Equation 10]**

*D*_b_ = [<=0.146, 0.15, 0.181, 0.199, 0.234, 0.251, 0.351, 0.429, 0.455, >=0.507]

**[Supplementary Equation 11]**

*θ*_r_ = [0.193, 0.371, 0.328, 0.385, 0.31, 0.207, 0.3, 0.31, 0.29, 0.34] **[Supplementary Equation 12]**

*n* = [1.348, 1.871, 1.533, 1.659, 1.753, 1.552, 1.53, 1.53, 1.53, 1.64]

**[Supplementary Equation 13]**

α = [0.114, 0.093, 0.066, 0.069, 0.033, 0.032, 0.22, 0.23, 0.21, 0.15]

**[Supplementary Equation 14]**

**For non-peatland mineral soils**

*θ*_r_ = 0.01*(22.733 - 16.4 * SAND + 0.0235 * CEC - 0.831 * PH + 18.0 * CLAY^2^ + 26.0 * SAND * CLAY)^9^ **[Supplementary Equation 15]**

$n= e^{0.01*(62.986 - 83.3*CLAY - 52.9 * 0.5*OM + 0.593*PH + 70.0 * {CLAY}^{2}-140*SAND*SILT)}$^9^

**[Supplementary Equation 16]**

$\alpha= e^{0.01*(-2.294 - 352.6 * SILT + 244.0 * 0.5 * OM - 0.0076 * CEC - 11.331 * PH + 190.0 * {SILT}^{2})}$^9^

**[Supplementary Equation 17]**

*θ*_fc_ = *θ*_r_ + (*θ*_s_ - *θ*_r_) * [1.0 + (α * *ψ*_fc_)^n^]^-m^  **[Supplementary Equation 18]**

*θ*_wp_ = *θ*_r_ + (*θ*_s_ - *θ*_r_) * [1.0 + (α* *ψ*_wp_)^n^]^-m^ **[Supplementary Equation 19]**

**For both peat and mineral soils**

PTDS = 1.3 – 2.66 * (1.0 – OM) **[Supplementary Equation 20]**

*θ*_s_ = 1.0 – *D*_b_/PTDS **[Supplementary Equation 21]**

λ = 3.0 – [ln(*θ*_fc_) – ln(*θ*_wp_)] / [ln(*ψ*_wp_) – ln(*ψ*_fc_)] **[Supplementary Equation 22]**

*K*_S_ = 1930.0 * (*θ*_s_ - *θ*_fc_)^λ^ **[Supplementary Equation 23]**

Where, θ_v_ = volumetric water content (m^3^ m^-3^), θ_r_ = residual water content (m^3^ m^-3^), θ_s_ = water content at saturation (m^3^ m^-3^), θ_fc_ = water content at field capacity (m^3^ m^-3^), θ_wp_ = water content at permanent wilting point (m^3^ m^-3^), ψ = soil water metric potential (-MPa), ψ_fc_ = soil water metric potential at field capacity (set at -0.033 MPa or 330 cm for mineral soils and -0.01 MPa or 100 cm for peat soils), ψ_wp_ = soil water metric potential at permanent wilting point (set at -1.5 MPa or 15000 cm), n = slope parameter of van Genuchten soil moisture retention curve (-), m = 1-1/n, α = parameter influencing the inflection point of the sigmoidal van Genuchten soil moisture retention curve (-MPa^-1^ or cm^-1^), S_e_ = relative saturation (-), K_r_ = relative hydraulic conductivity (-), K_s_ = saturated hydraulic conductivity (mm hr^-1^), K_unsat_ = unsaturated hydraulic conductivity (mm hr^-1^), S_c_ = relative saturation at air entry point (-), ψ_e_ = soil water metric potential at air entry point (set at -0.00058 MPa)^8^, *D*_b_ = dry bulk density (Mg m^-3^), PTDS = particle density (Mg m^-3^), λ = slope to estimate saturated hydraulic conductivity (-), OM = soil organic matter fraction, SAND = soil sand content fraction, SILT = soil silt content fraction, CLAY = soil clay content fraction, PH = soil pH, CEC = soil cation exchange capacity (meq 100g^-1^ or cmol_(c)_ kg^-1^)

Hydrology in ecosys model is not simulated in isolation, rather it is simulated as an integral part of a holistic and interlinked soil-plant-microbe-atmosphere carbon, nutrient, heat and water balance. So, hydrology in ecosys is affected by and affects other cycles such as plant water relations, plant CO_2_ fixation, peat and mineral soil aeration, decomposition, and degradation. Peatland water table depth can be affected by peat subsidence, which is driven by peat decomposition due to water table drawdown by dry weather and artificial drainage. Although peat decomposition is explicitly modelled within the ecosys model, the current version of the hydrology sub-model of ecosys did not simulate the effect of subsidence on modelled water table depth. Instead, we assumed the peat surface to be static throughout our simulation in this study.

**1.2. Site measurements for peatland water table depths**

The *ecosys* tropical peatland hydrology model was validated against WTD measurements over six tropical peatland sites across Riau province of Sumatra (Supplementary Fig. 6). Those sites represented dominant land use and land cover types and land management (e.g. drained vs. undrained) across tropical peatlands in Riau (Supplementary Fig. 6). Four of the WTD measurement sites were not affected by artificial drainage. Those sites were ‘Pristine Peat Swamp’, ‘Zamrud Peat Swamp’, ‘*Acacia*’ and ‘Oil Palm and Rubber’. The remaining two sites – ‘Bintangur Peat Swamp (drained)’ and ‘*Acacia*/*Eucalyptus* (drained)’ were drained peatlands^10^. WTD was measured by installing polyvinyl chloride (PVC) pipes at the six measurement sites. These measurements were carried out in 2010 over the two drained sites and in 2018 over the undrained sites representing ranges of weather conditions across tropical peatlands in Riau. The WTD measurements were carried out at every 15 minutes over the drained sites and at every 10 minutes over the undrained sites by using automated WTD sensors installed with the PVC pipes. The lengths of WTD pipes were kept between 0.75 and 1.0 m above the ground surface in the two undrained sites - ‘*Acacia*’ and ‘Oil Palm and Rubber’. The above ground lengths of the WTD pipes were between 0.15 and 0.25 m in the other four sites. The WTD measurements at every time-step (10 or 15 minutes) were processed to smooth out any noises due to sensor calibration and then averaged up to hourly WTD measurements. Hourly precipitation was measured from November 2009 to April 2011 at a meteorological station close to the ‘*Acacia*/*Eucalyptus* (drained)’ site^10^. Hourly precipitation was also measured from March to November 2018 at meteorological stations over the four undrained sites. Besides, peat dry bulk density was measured at different depths over the four undrained sites. Peat depths at the six WTD measurement sites varied between 3 and 8 m.

**1.3. Site-level peatland hydrology modelling**

A site-level *ecosys* hydrology model run was set up for each of the WTD measurement sites described above (Supplementary Fig. 6). Each of these site-level modelled grids were of 150 m x 150 m in dimensions to represent the size of the smallest tropical peatland management units (usually 2 ha). Modelled site-level peat soil profiles were 6 m in depth, each of which was subdivided into 15 vertical layers for improved resolution in modelling vertical and lateral water fluxes (Supplementary Table 5). These modelled peat layers were built by using peat soil properties measured at different depths either at the sites or reported in literature for similar sites (Supplementary Table 5). Peat moisture contents at saturation (θ_s_) was calculated from the model inputs of dry bulk density at each layer (Supplementary Equations 20-21). Model parameters for peat moisture retention properties such as, residual peat moisture contents (θ_r_), slopes (*n*) and the inflection points (*α*) of the sigmoidal moisture retention curves were estimated internally at the start of each model run by fitting four points on a moisture release curve, which includes θ_s_, metric potential at inflection point (ψ_in_), and peat moisture contents at field capacity (θ_fc_) and permanent wilting point (θ_wp_)^4^ (Supplementary Table 5) (Supplementary Equations 1-3). Model inputs for ψ_in_, θ_fc_, and θ_wp_ at each depth of a model peat profile were estimated from moisture retention data from published literature^11,12,13^ for different peat depths across different land use, land cover and land management in Sumatra and Kalimantan (Supplementary Equations 10-14). Model input for saturated hydraulic conductivity (*K*_s_) for each layer was estimated from a published pedo-transfer function^14^ (Supplementary Table 5) (Supplementary Equations 20-23).

Each of the six site-level model runs included a spin-up run for 54 years using repeated weather sequences from 2008 to 2018 which was continued to a simulation run from 2008 to 2018. The spin-up runs allowed the modelled mass and energy balances to attain dynamic equilibria so that the seasonal variations of hydrology in the simulation runs were only affected by model drivers not by any boundary conditions at the start of the spin-up runs. Model inputs of hourly shortwave radiation, air temperature, relative humidity and wind speed during 2008-2018 were derived from ECMWF’s ERA5. Model inputs of hourly precipitation were derived from Climate Hazards Group InfraRed Precipitation with Station (CHIRPS) dataset by dividing the daily CHIPRS precipitation into equally distributed 24-hourly precipitation values for each day. The CHIRPS precipitation data was replaced with hourly precipitation measured over the sites for the periods where hourly site measurements were available. Throughout the spin-up and simulation runs, the model lateral boundary WTDx was set as 0.6 m below the surface for undrained and 1.0 m below the surface for drained sites (Supplementary Table 5). For the ‘*Acacia*/*Eucalyptus* (drained)’ site, the WTDx was reset as 0.2 m shallower (WTDx = 0.8 m below the surface) from June to December 2010 to simulate maintenance of higher canal water level by using WTD gate^10^ (Supplementary Table 5). Each modelled grid was seeded by plant functional types (PFTs) that represented eco-physiological characteristics of dominant vegetation at the site (Supplementary Table 5).

Modelled outputs of hourly WTD values were validated against available hourly observed WTD values. Model performance in simulating hydrologic dynamics was evaluated by correlation coefficient (*r*), root mean squared error (RMSE), and Willmott’s index of model agreement (*d*)^15,16^. Values of 1.0 for *r*, 0.0 for RMSE and 1.0 for *d* between modelled and observed hourly WTD for a given site with a given weather condition would mean a perfect simulation of hydrologic dynamics. These site-level model validations allowed us to assess whether the model algorithms, model inputs for peat physical and hydrologic parameters and the various assumptions we made regarding modelling lateral boundary conditions were sufficiently good enough to enable us to confidently simulate seasonal variations in hydrology across tropical peatlands with a high degree of accuracy.

**1.4. Non-peatland hydrology modelling**

Hydrologic variations in non-peatland grid cells were simulated following the same protocols as described in the previous section for the peatland grids. As in the peatland grid cells, van-Genuchten soil moisture retention parameters for non-peatland mineral soils were estimated internally at the start of each model run by optimizing θ_s_, ψ_in_, and θ_fc_ and θ_wp_ (Supplementary Table 5) (Supplementary Equations 1-3). Model inputs for ψ_in_, θ_fc_, θ_wp_ and *K*_s_ at each vertical layer of a model peat profile were estimated from pedo-transfer functions available for tropical mineral soils in published literature^11,14^ (Supplementary Table 5) (Supplementary Equations 15-23). One significant difference in modelling non-peatland hydrology with respect to peatland hydrology was in setting the depths of the external water table (WTDx) as lateral boundary conditions. Unlike shallower WTDx in the peatland grids, the non-peatland WTDx was set to the bottom of each non-peatland grid cell (WTDx = 6 m below the surface) to allow discharge throughout the vertical soil profile to mimic better drainage of mineral soils as opposed to peat soils. The modelled WTD in non-peatland grids was, however, modelled at the end of each time-step, like peatland grid cells, as the depth below which modelled air-filled porosity was zero."

**2. Supplementary Discussion**

Riau has a total area of 87,024 km^2^, about 44% of which is occupied by tropical peatlands with peat accumulations of 0.5 m or deeper (Fig. 1). However, in 2015, only about 8% of the total peatlands in Riau remained under pristine peat swamp forest. Remaining peatlands in Riau were either degraded by logging (~17%) or converted for industrial plantations (~30%) and small holders’ farmlands (~36%) (Fig. 1)^17^. Conversion of pristine peat swamp forests into other land uses can significantly alter hydrology and water balance of those tropical peatlands by changing vegetation composition, micro-meteorology and water table depth through artificial drainage^1,2,18^. Interannual variations in seasonality in precipitation (e.g., drought year vs. regular year) can also control the seasonality in hydrology across both pristine and converted tropical peatlands^1,2,18^. We tested the predictive capacity of tropical peatland hydrology model *ecosys* (Supplementary Fig. 1) in simulating seasonal variation in WTD over 6 site-years under different weather conditions, and land uses and land management (drained vs. undrained) across peatlands in Riau (Supplementary Fig. 6).

*Ecosys* could successfully simulate observed seasonal variations in WTD over a range of precipitation from 2373 to 3162 mm yr^-1^ as indicated by high correlation coefficients (r) (>0.75) and Willmott’s index of model agreement (d) (>0.8) and low root mean squared errors (RMSE) (~0.1 m) between modelled and observed hourly WTD (Supplementary Figs. 6-7). During the rainy seasons, precipitation exceeded simulated evapotranspiration that caused net vertical water recharge, which raised modelled WTD and drove lateral discharge from the modelled peat soil profiles^4^ (Supplementary Fig. 7). The rate of lateral discharge was controlled by moisture holding capacities and hydraulic conductivities of different vertical modelled peat soil layers and the hydraulic gradient between WTD within the modelled peat soil profile and an external boundary WTD termed as WTDx^4^ (Supplementary Fig. 1). A WTDx was a pre-defined lateral boundary condition, which mimicked the WTD of an adjacent watershed or a canal with reference to the surface of a modelled peat or mineral soil profile^4,18^. Modelled WTD was the vertical point within a modelled peat soil profile below which the modelled peat soil layers were completely saturated. Sometimes during the rainy season, when the modelled peat soil profiles were entirely saturated, the WTD came up to or even above the surface of the modelled peat soil profiles, in which case the WTD was the depth of standing water above the surface (Supplementary Fig. 7). The depth of the standing water above modelled surface was controlled by the rate of surface run-off that was driven by the hydraulic head, surface roughness and the slope of the grid cell^4^. With the onset of the dry season, lateral discharge exceeded the vertical recharge (precipitation minus evapotranspiration), which caused drawdown of modelled WTD^4^ (Supplementary Fig. 7). Recession of modelled WTD reduced the hydraulic gradient between WTD and WTDx, which gradually slowed down lateral discharge towards the end of the dry season (Supplementary Fig. 7) ^1,18^. Beside the seasonality in vertical and lateral water balance, both modelled and observed WTDs over those tropical peatland sites were significantly altered by artificial drainage (Supplementary Figs. 6-7)^1,2,18^. Unlike over undrained sites, WTD in drained sites never came up to or above the ground surface during the rainy seasons (Supplementary Figs. 6-7). Instead, the WTD in the two drained sites always remained about 40 cm or deeper from the ground surface even in the rainy season^1,18^. Deeper WTD simulations over drained sites than in undrained sites was achieved in *ecosys* by pre-setting deeper WTDx in drained simulations than in undrained simulations to model artificial drainage^4^ (Supplementary Table 5).

WTD and near surface soil moisture followed the similar seasonal patterns as the peatland hydrology. During the rainy season in 2015, when precipitation exceeded evapotranspiration, modelled WTD rose, and the modelled soil became wet (Supplementary Figure 4c). As the dry season approached, modelled lateral drainage and evapotranspiration exceeded vertical recharge through precipitation, which caused modelled WTD to recede to a deeper position and near surface soil desiccation until the rainy season arrived at the end of the dry season causing increased vertical recharge and hence a shallower WTD and wetter modelled near surface soil (Supplementary Figure 4c). While the comparative lack of site-level measurements of soil moisture contents and WTD did not allow a direct and detailed benchmarking of modelled hydrologic dynamics in non-peatland grids (in contrast to the situation for site level validation in peatland cells), we nonetheless consider our approach to hydrologic modelling in non-peatland grid as generally accurate because of the close match between modelled soil moisture and SMAP soil moisture observations (Supplementary Figure 4).

Although our hydrologic modelling was able to simulate variations in observed water table depth and near surface soil moisture retention from available site measurements and SMAP satellite data products, uncertainties remained. One key uncertainty was the dearth of available data and pedo-transfer functions to estimate key hydrologic properties to drive the hydrology model. We are unaware of any published pedo-transfer function specifically developed for tropical peatlands. As a pragmatic approach, we adapted a pedo-transfer function predominantly developed for mineral soils, to derive saturated hydraulic conductivities for each modelled peat layer (Supplementary Equations 22-23). We rigorously tested the pedo-transfer function by comparing site level hourly modelled peatland WTD against observed WTD. The modelled hourly peatland WTD across contrasting peatland sites under a range of precipitation and drainage conditions demonstrated a very strong correlation (all correlation coefficients > 0.8) and small errors (root mean squared error < 0.1 m) with hourly observed WTD data (Supplementary Figure 7). Consequently, we consider our adaption of this mineral soil pedo-transfer function as adequate for the purposes of the present study to simulate tropical peatland hydrology.

Another potential source of uncertainty in peatland hydrology modelling was the assumption of vertical and lateral saturated hydraulic conductivity being the same for each modelled layer. Tropical peatlands are bog peatlands which are known to be anisotropic and can have higher lateral preferential flow especially in the top less decomposed fibric layers ^19,20^. To test the adequacy of the hydraulic equality assumption, we ran four parallel simulations at each of the 6 peatland sites (Supplementary Figure 6). Each simulation assumed lateral to vertical saturated hydraulic conductivity ratios of 0.5, 1.0, 1.5, and 2.0. For all the six sites, the root mean squared errors between modelled versus observed hourly WTD were at least 0.2 m less in the simulation using the ratio 1.0 than the other three scenarios. That is why we reported the site validation results for the scenario using equal vertical and lateral conductivities for each modelled layer (Supplementary Figure 7) and extended the same assumptions for Riau-wide hydrologic simulations.

Our hydrologic modelling was achieved by simulating vertical and lateral fluxes as a function of peat and mineral soil hydrologic properties. The vertical water fluxes through the modelled soil profile were constrained by vertical hydraulic conductivity as influenced by peat or mineral soil moisture retention properties (Supplementary Figure 1) (Supplementary Equations 1-23). For instance, higher saturated hydraulic conductivities in peatland grids compared to non-peatland grids (Supplementary Figure 18) contributed to higher modelled infiltration, lower modelled surface runoff (Supplementary Figure 5) and hence shallower modelled WTD in peatlands than in no-peatland areas (Figure 2) (Supplementary Figure 4). The soil moisture retention was also controlled by the total porosity and soil moisture contents at saturation (Supplementary Figure 1) (Supplementary Equations 1-23). Higher soil water contents at saturation for peatland grids (Supplementary Figure 14) enabled *ecosys* to correctly simulate higher soil water contents in peatland grids than in non-peatland grids (Figure 2) (Supplementary Figure 4). Modelled lateral water fluxes were driven by lateral hydraulic gradient as controlled by the modelled WTD and the external boundary WTD (WTDx) (Supplementary Figure 1) and constrained by lateral hydraulic conductivity. Shallower-set WTDx in undrained peatland site enabled *ecosys* to correctly simulate shallower WTD compared with drained peatland sites (Supplementary Figure 7). Similarly, shallower WTDx in peatland grids contributed to the correct simulation of shallower WTD than in non-peatland grids (Figure 2) (Supplementary Figure 4).


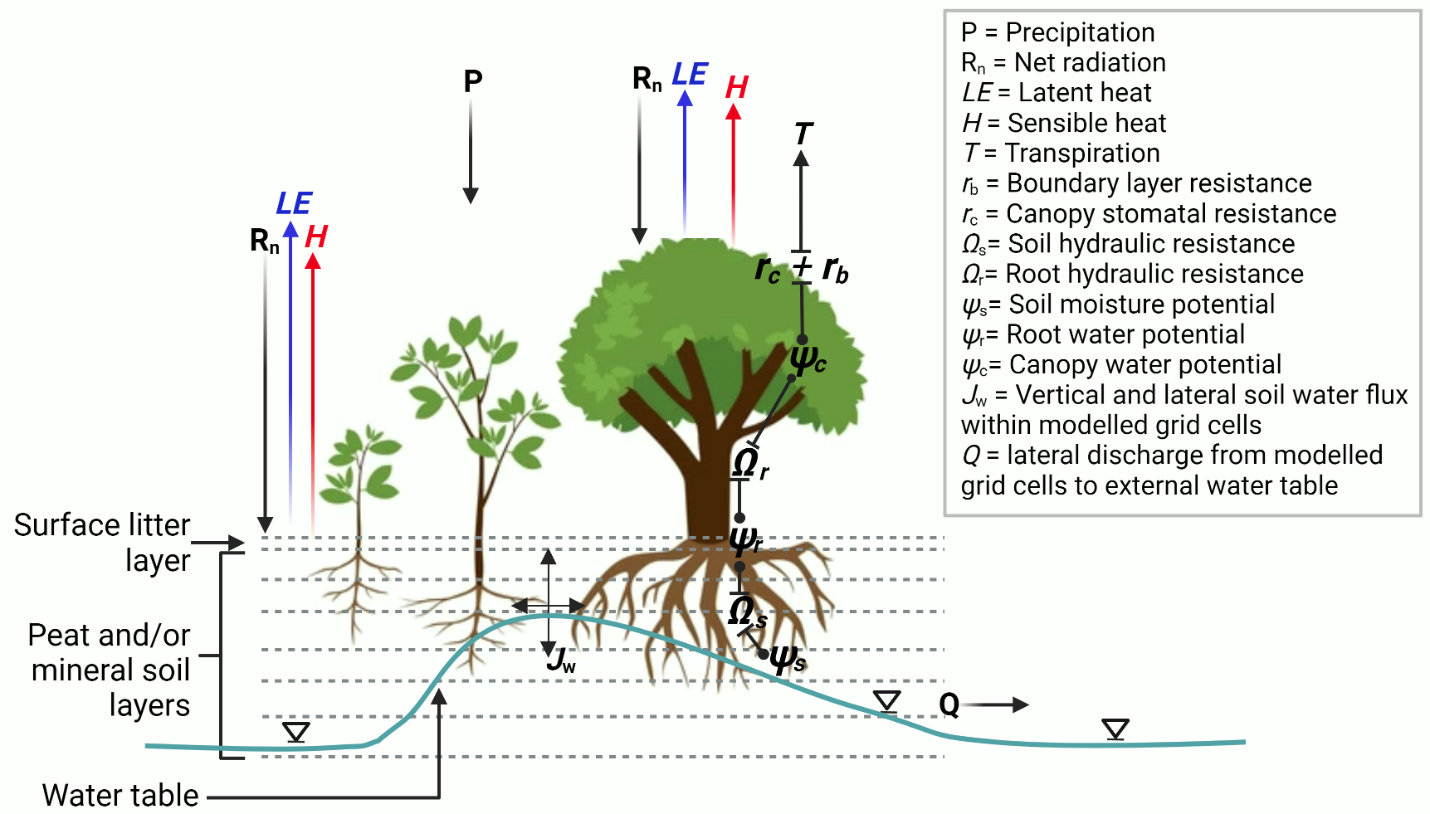


**Supplementary Figure 1. Schematic diagram of key eco-hydrology process simulations in *ecosys*.** The figure was drawn by using bioRENDER online (https://biorender.com/).


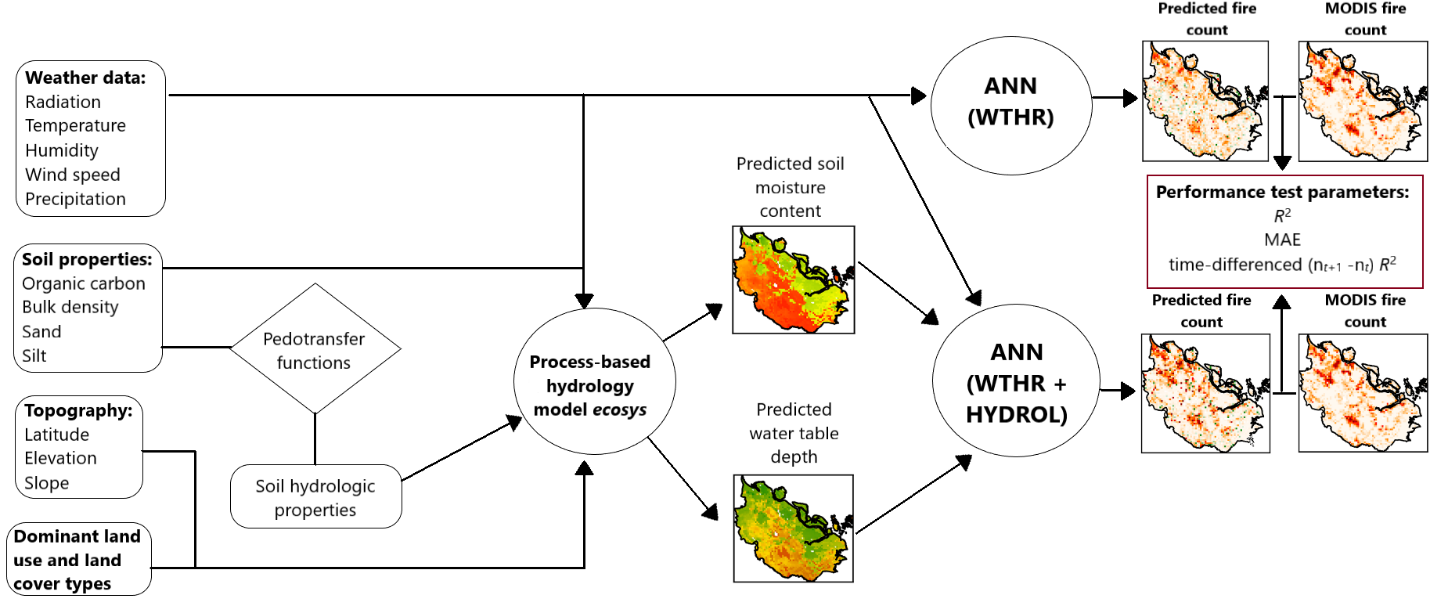


**Supplementary Figure 2. Modelling workflow of including hydrological dynamics into seasonal fire forecasting across Riau province of Sumatra, Indonesia.** ANN (WTHR + HYDROL) = artificial neural network model featured by both weather and hydrology variables, ANN (WTHR) = artificial neural network model featured by only weather variables, *R*^2^ = coefficient of determination between predicted and Moderate Resolution Imaging Spectroradiometer (MODIS) active fire count, MAE = mean absolute error between predicted and MODIS active fire count, *n* = active fire count, and *t* = model time-step (two weeks). The maps were created by using Julia Programming Language version 1.6.7 (<https://julialang.org/downloads/>).


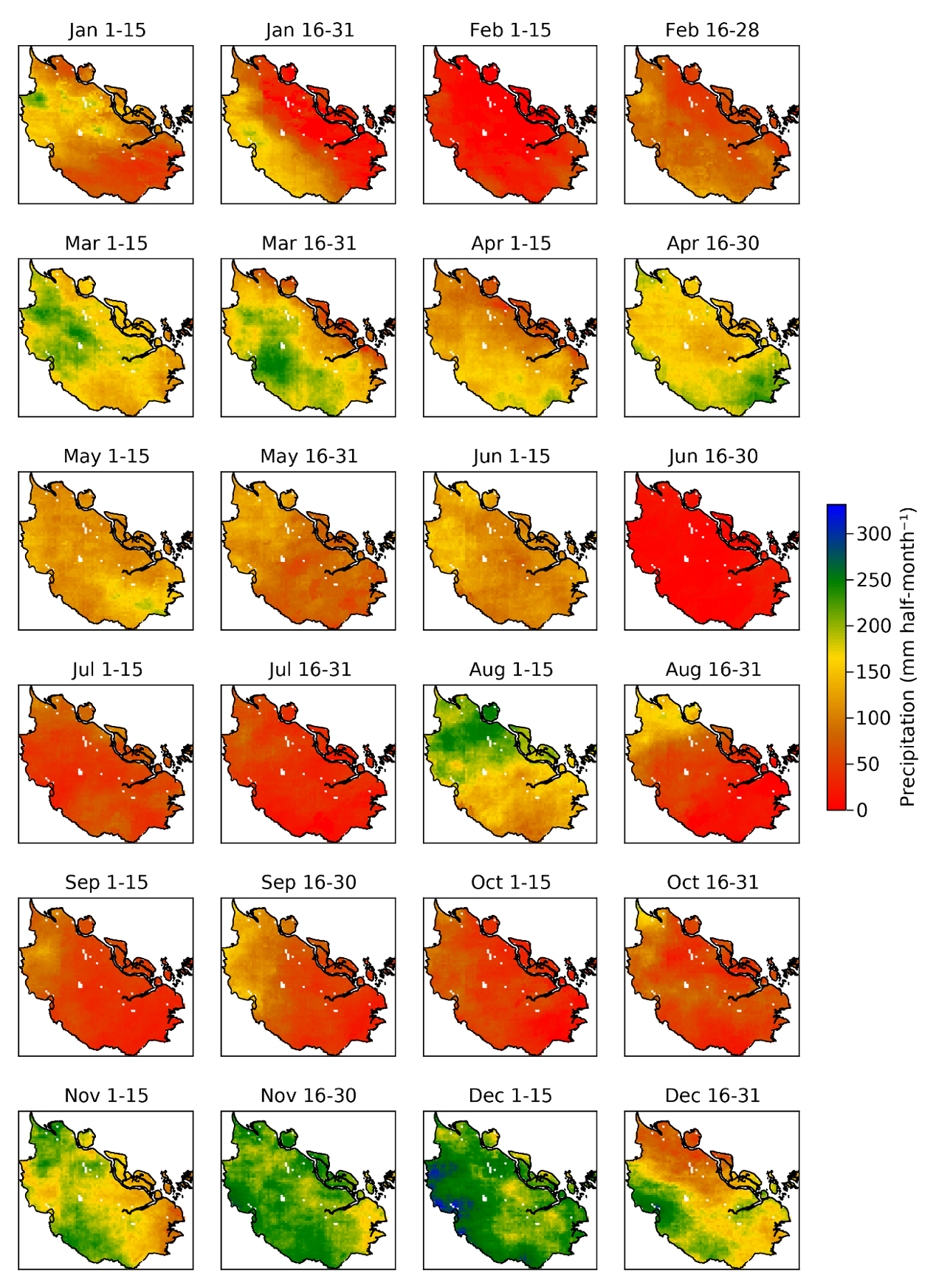


**Supplementary Figure 3. Half-monthly precipitation during 2015 across the Riau province of Sumatra, Indonesia**. The precipitation data was obtained from Climate Hazards Group InfraRed Precipitation with Station (CHIRPS) dataset (Supplementary Table 2). The maps were created by using Julia Programming Language version 1.6.7 (<https://julialang.org/downloads/>).


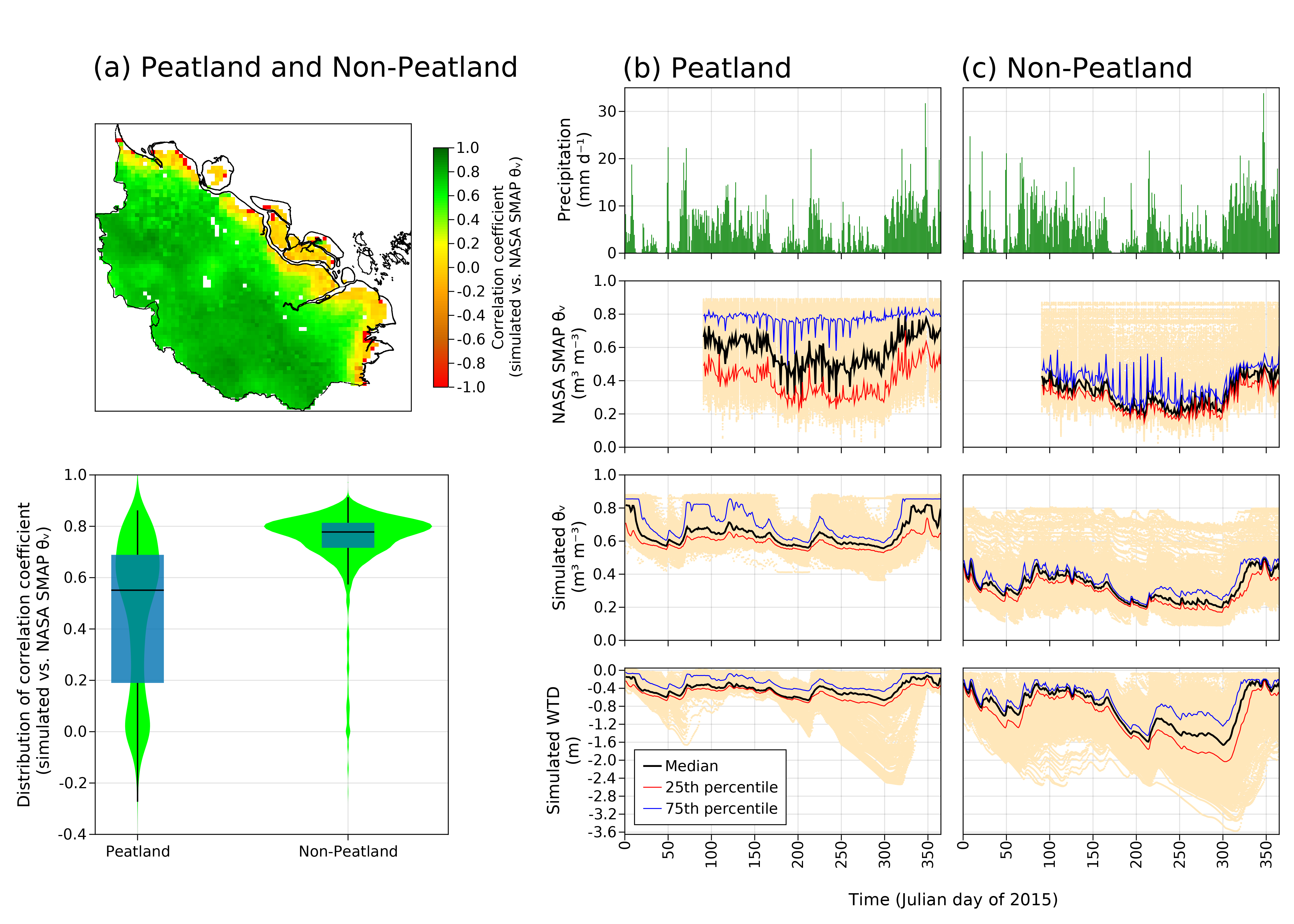


**Supplementary Figure 4. Validating and explaining spatial simulation of seasonal variations in water table depth (WTD) and near-surface peat and mineral soil moisture contents.** **(a)** Spatially distributed temporal correlation and the distribution of correlation coefficients between daily simulated and National Aeronautics and Space Administration (NASA) Soil Moisture Active Passive (SMAP) near-surface (0-0.05 m) soil moisture contents (𝜃_v_), as represented on the map and described by the vertical probability distribution curves and box and whisker plots; and average daily precipitation, daily SMAP and simulated near-surface soil moisture contents (𝜃_v_) and daily simulated water table depth (WTD) during 2015 for **(b)** peatland and **(c)** non-peatland grids across Riau province, Sumatra, Indonesia. Negative water table depths are depths below the ground surface. The map in **(a)** was created by using Julia Programming Language version 1.6.7 (<https://julialang.org/downloads/>).


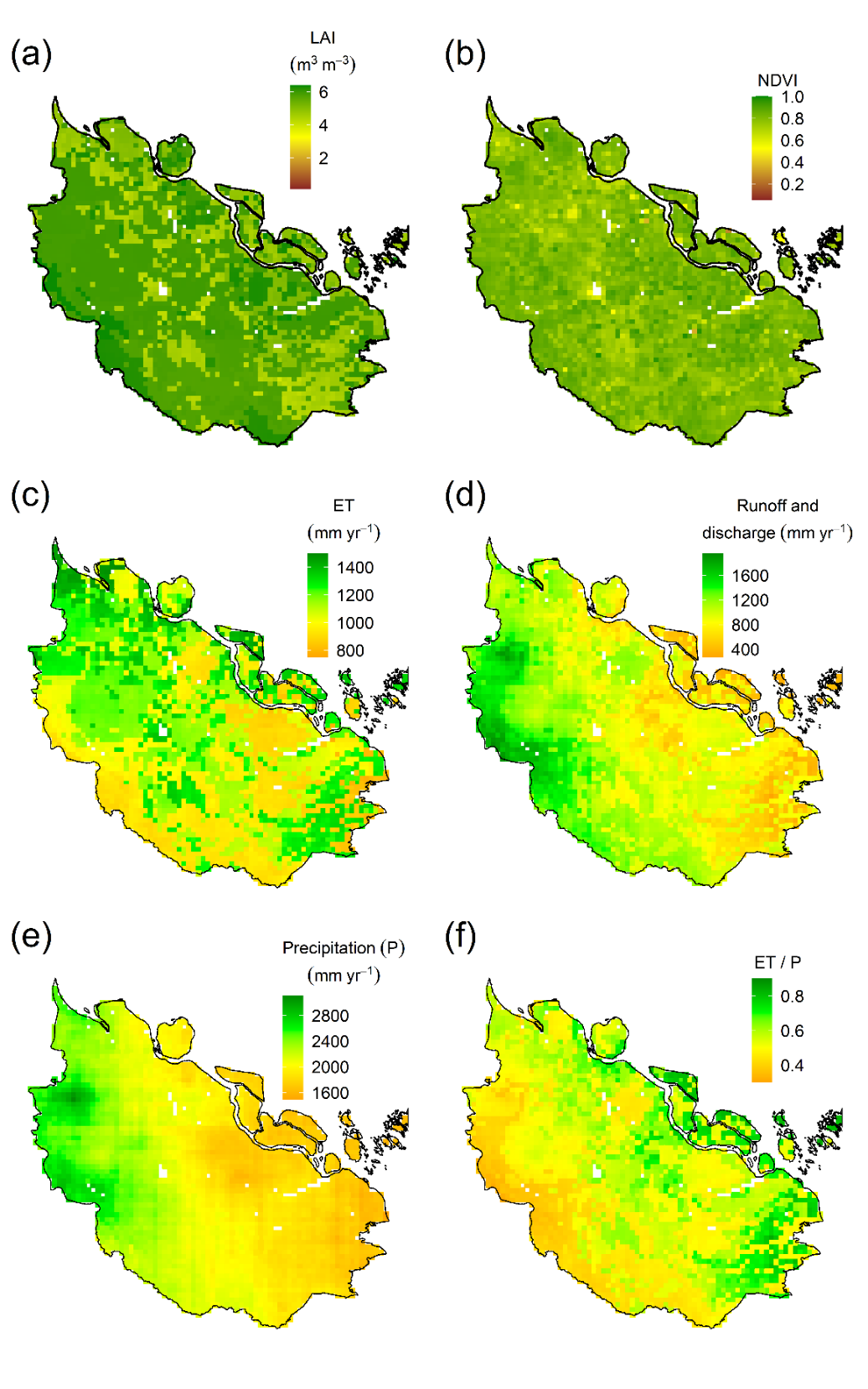


**Supplementary Figure 5. Modelled annual water balance during 2015 across the Riau province of Sumatra, Indonesia**. Spatial distribution of **(a)** mid-season (July 13 – July 28) simulated leaf area index (LAI) and **(b)** Moderate Resolution Imaging Spectroradiometer (MODIS) Normalized Difference Vegetation Index (NDVI), **(c)** simulated annual evapotranspiration (ET), **(d)** simulated annual sums of surface runoff and subsurface lateral discharge **(e)** annual CHIRPS precipitation (P), and **(f)** simulated ratio between ET and P. The maps in **(a-f)** were created by using R version 4.0.3 (https://cran.microsoft.com/snapshot/2021-01-17/bin/windows/base/).


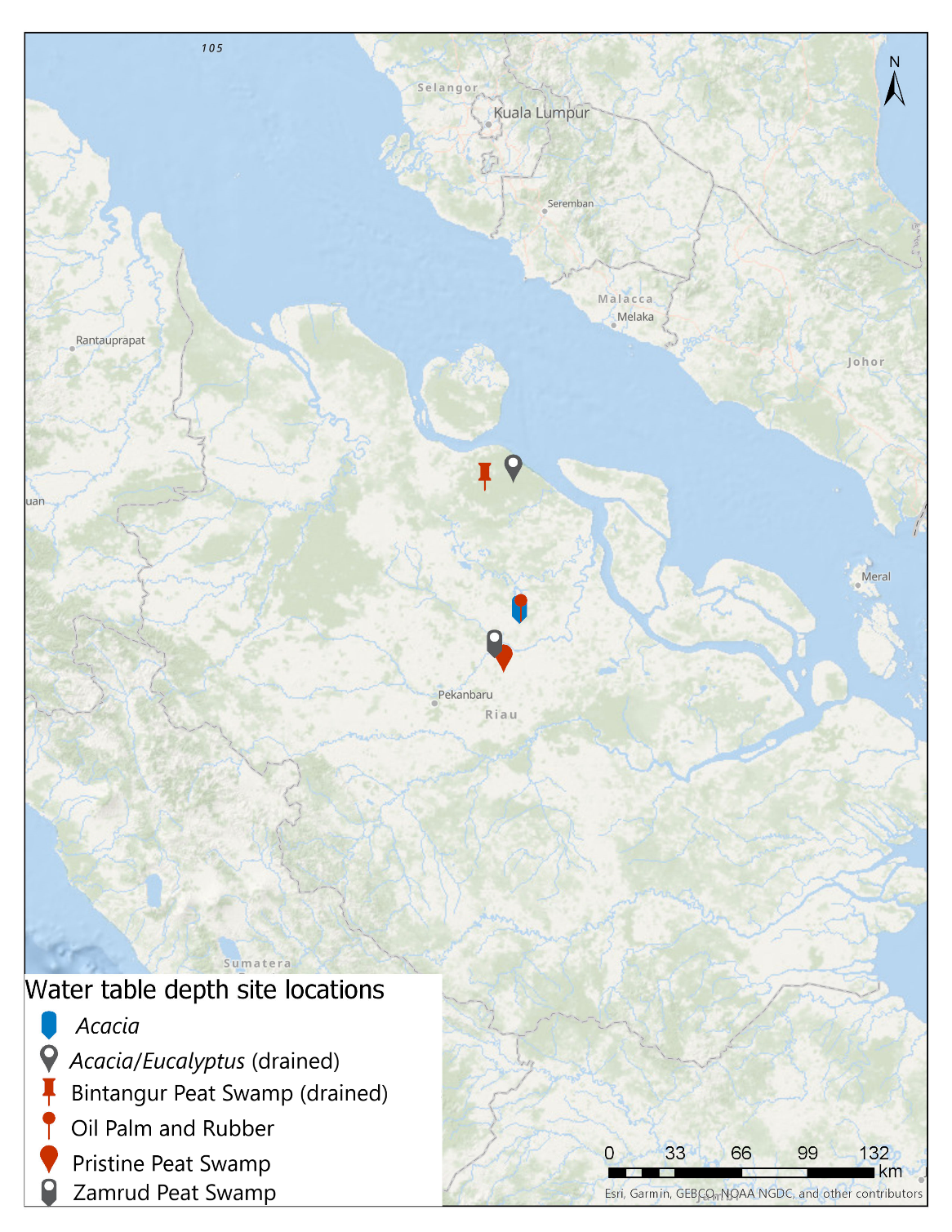


**Supplementary Figure 6. Locations of water table depth measurement sites across the Riau province of Sumatra, Indonesia**. The map was created by using ArcGIS Pro version 2.9 (<https://support.esri.com/en/Products/Desktop/arcgis-desktop/arcgis-pro/2-9#overview>).


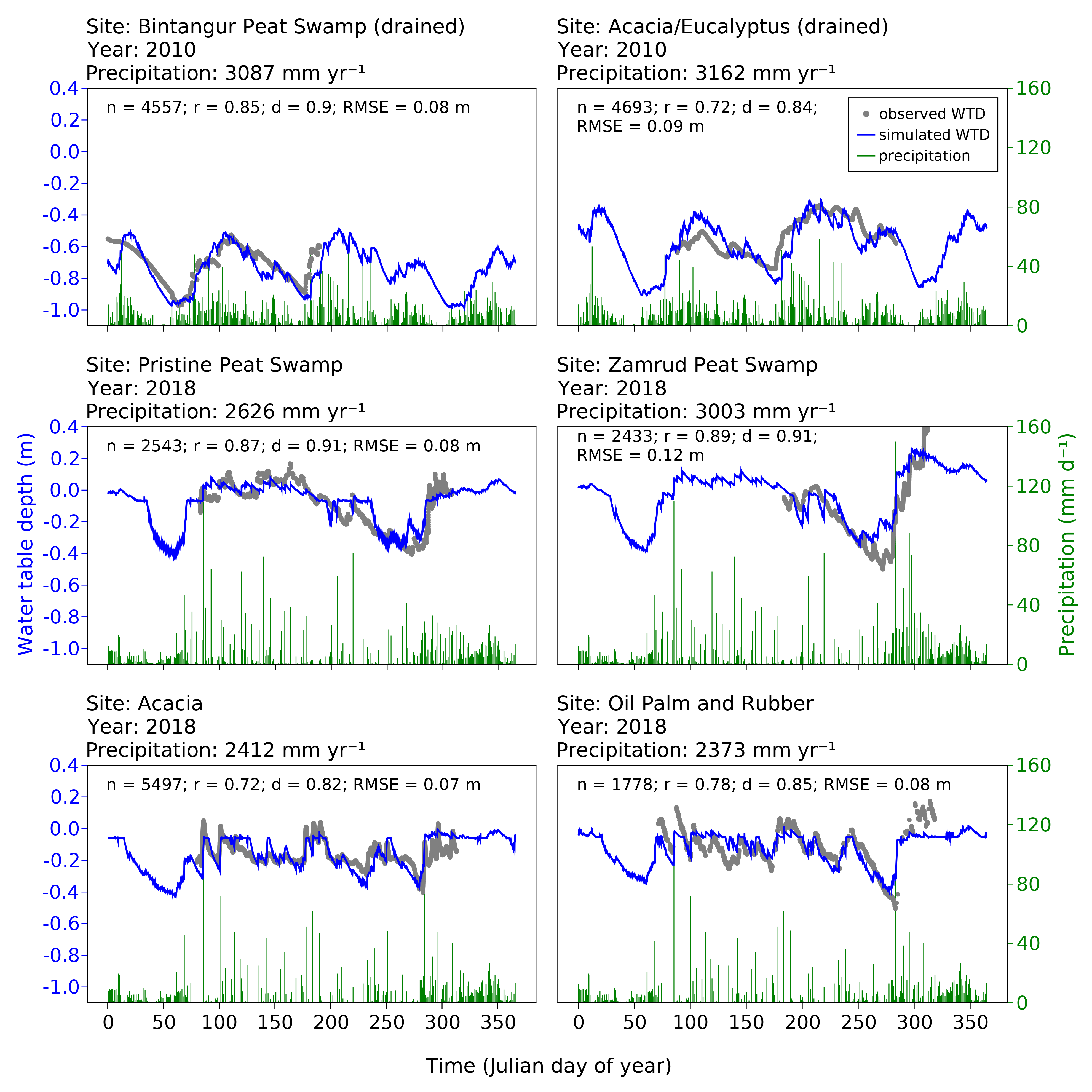


**Supplementary Figure 7.** **Modelling seasonal variations in water table depth across tropical peatland sites.** Daily precipitation, and hourly observed and simulated water table depth at six peatland sites under different land use, land cover and land management in different years across Riau province, Sumatra, Indonesia (see Supplementary Fig. 6 for locations of the sites). Negative water table depths are depths below the ground surface. n = number of observed hourly water table depth data; r = correlation coefficient; RMSE = root mean squared error; d = Willmott’s index of agreement between modelled and observed hourly water table depth.


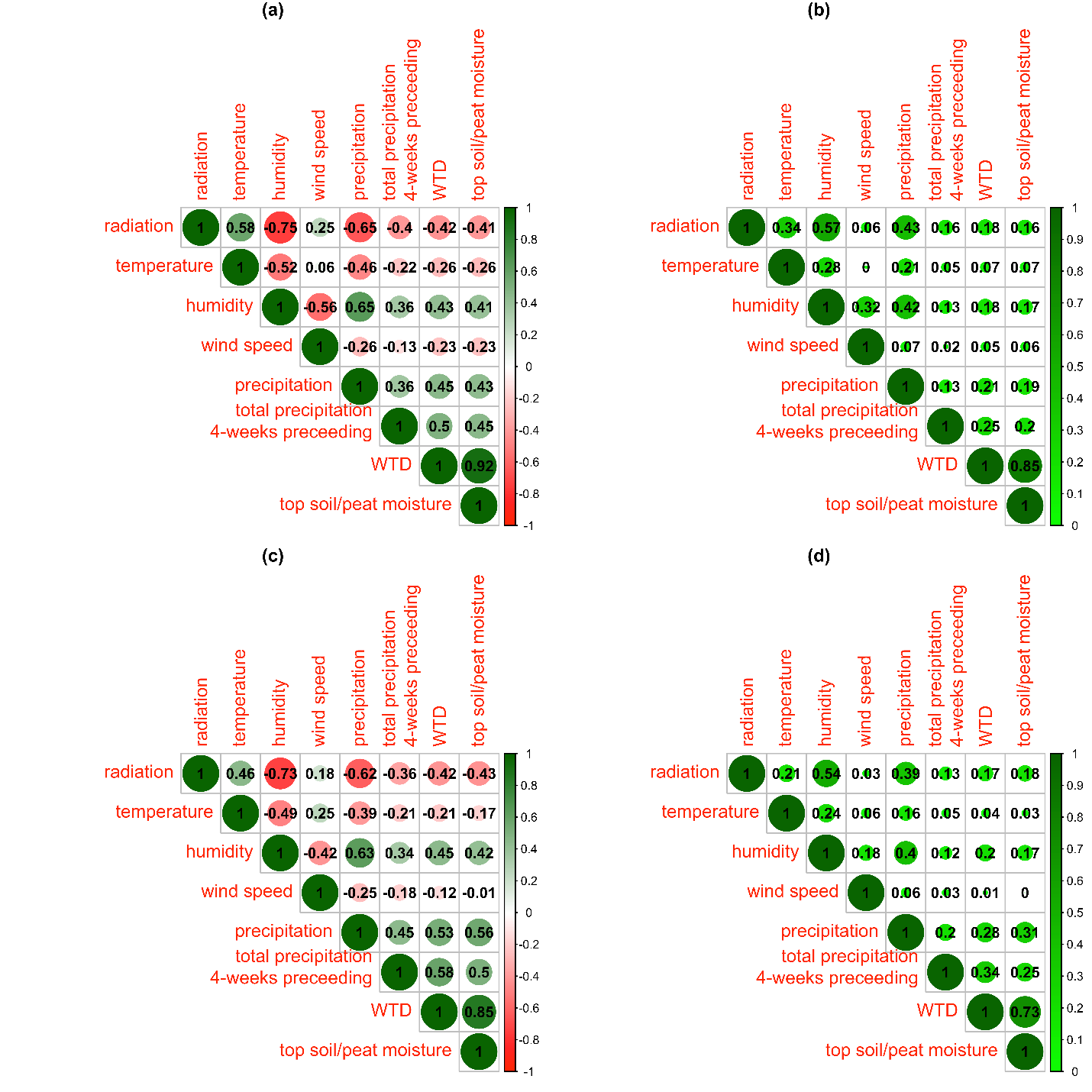


**Supplementary Figure 8.** **Autocorrelations between weather and hydrological predictor variables (features) in predicting active fire count.** **(a)** Correlation coefficient (r) for peatland grids (Supplementary Fig. 1) **(b)** coefficient of determination (R^2^) for peatland grids **(c)** r for non-peatland grids, and **(d)** R^2^ for non-peatland grids. All the features except the two precipitation variables were averaged for each two-week model time step. The two precipitation variables were –precipitation summed over each two-week time step and precipitation summed over 4-weeks leading to each model time step


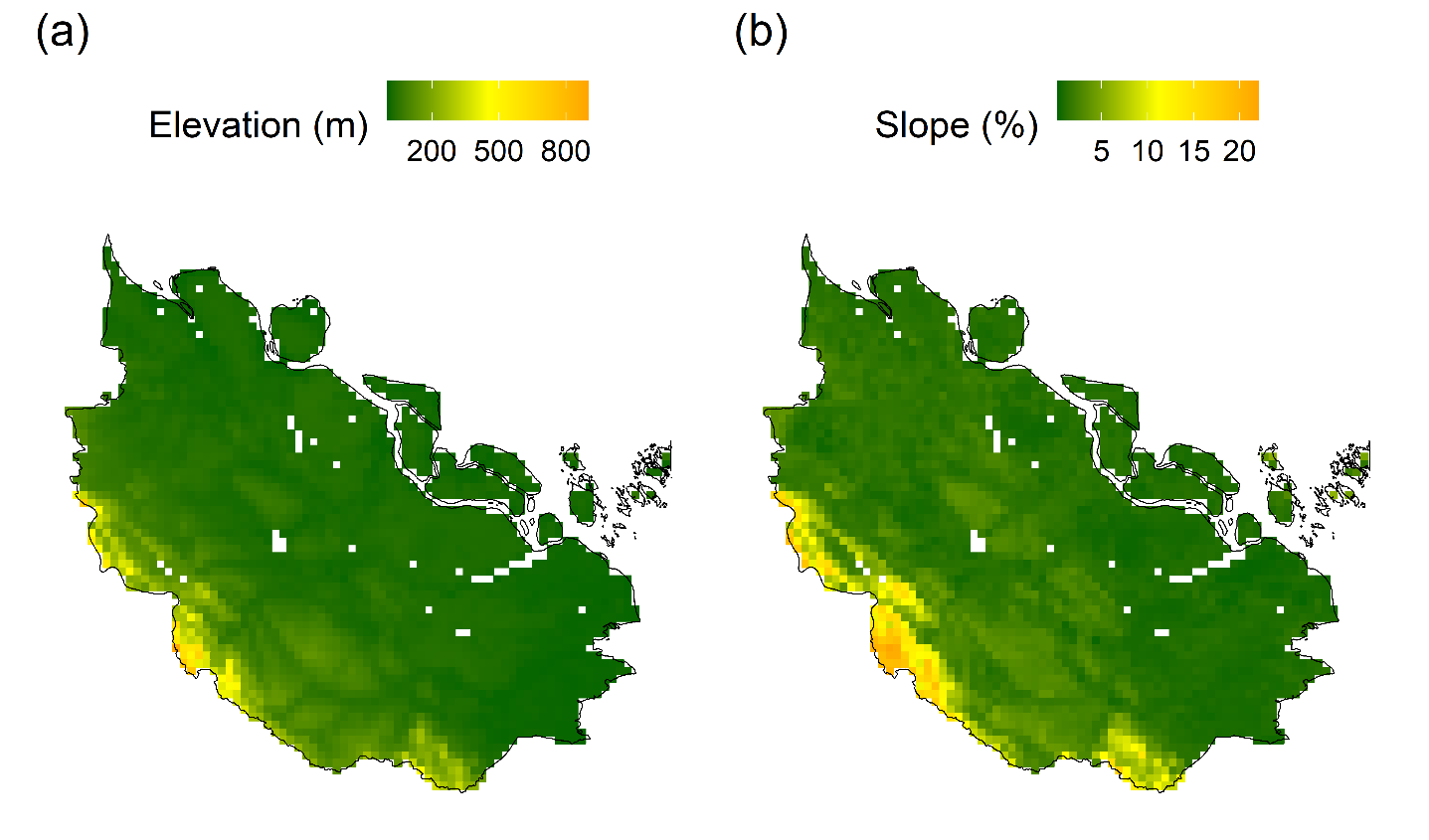


**Supplementary Figure 9. Model inputs of landform to simulate hydrologic dynamics across Riau province of Sumatra, Indonesia. (a)** Elevation and **(b)** slope^23^ (Supplementary Table 2). The maps in **(a-c)** were created by using R version 4.0.3 (https://cran.microsoft.com/snapshot/2021-01-17/bin/windows/base/).


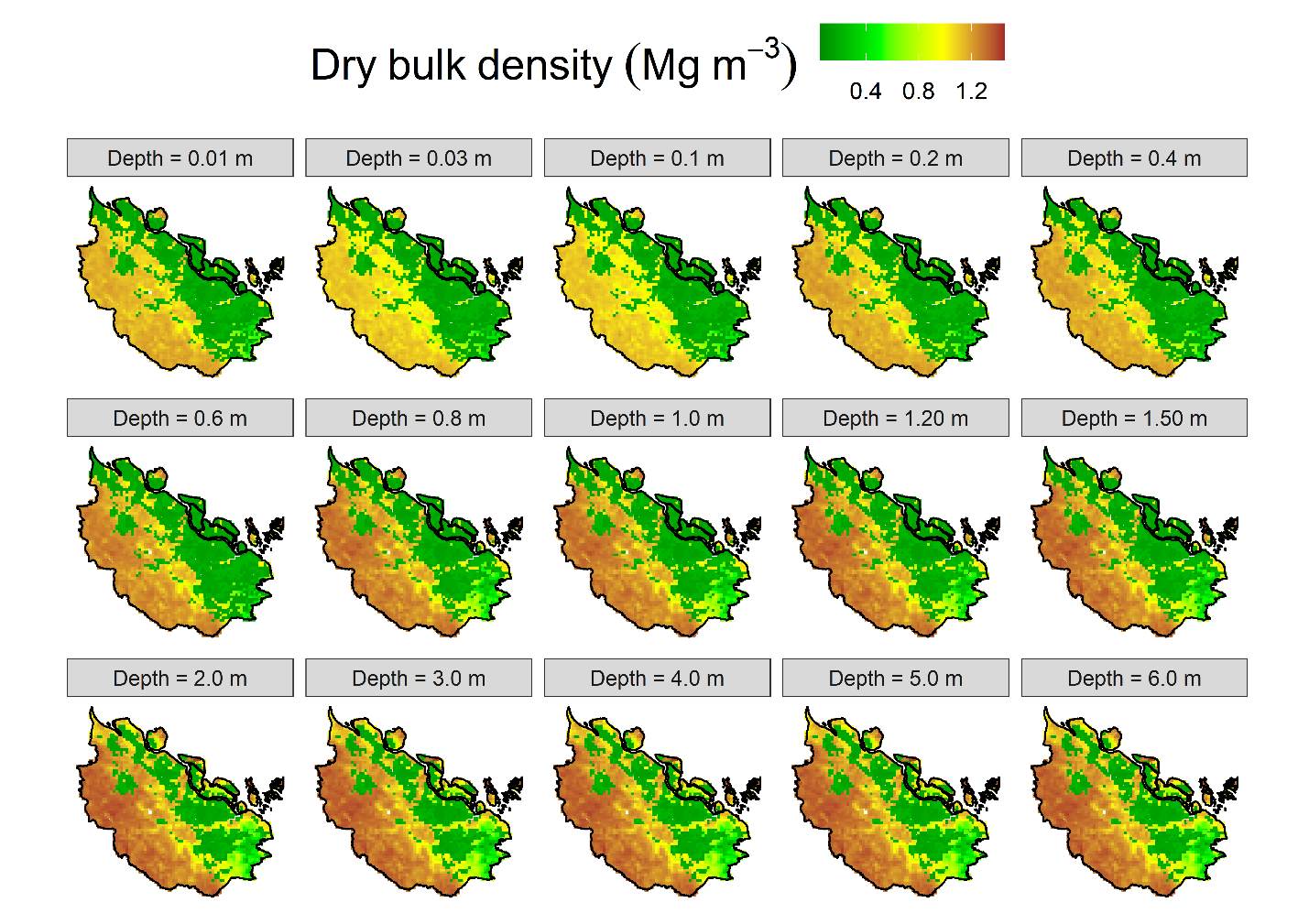


**Supplementary Figure 10. Model inputs of dry peat or mineral soil bulk density at different depths to simulate hydrologic dynamics across Riau province of Sumatra, Indonesia.** Values for the depths are depths to the bottom of each modelled vertical layer (Supplementary Table 2). The maps were created by using R version 4.0.3 (https://cran.microsoft.com/snapshot/2021-01-17/bin/windows/base/).


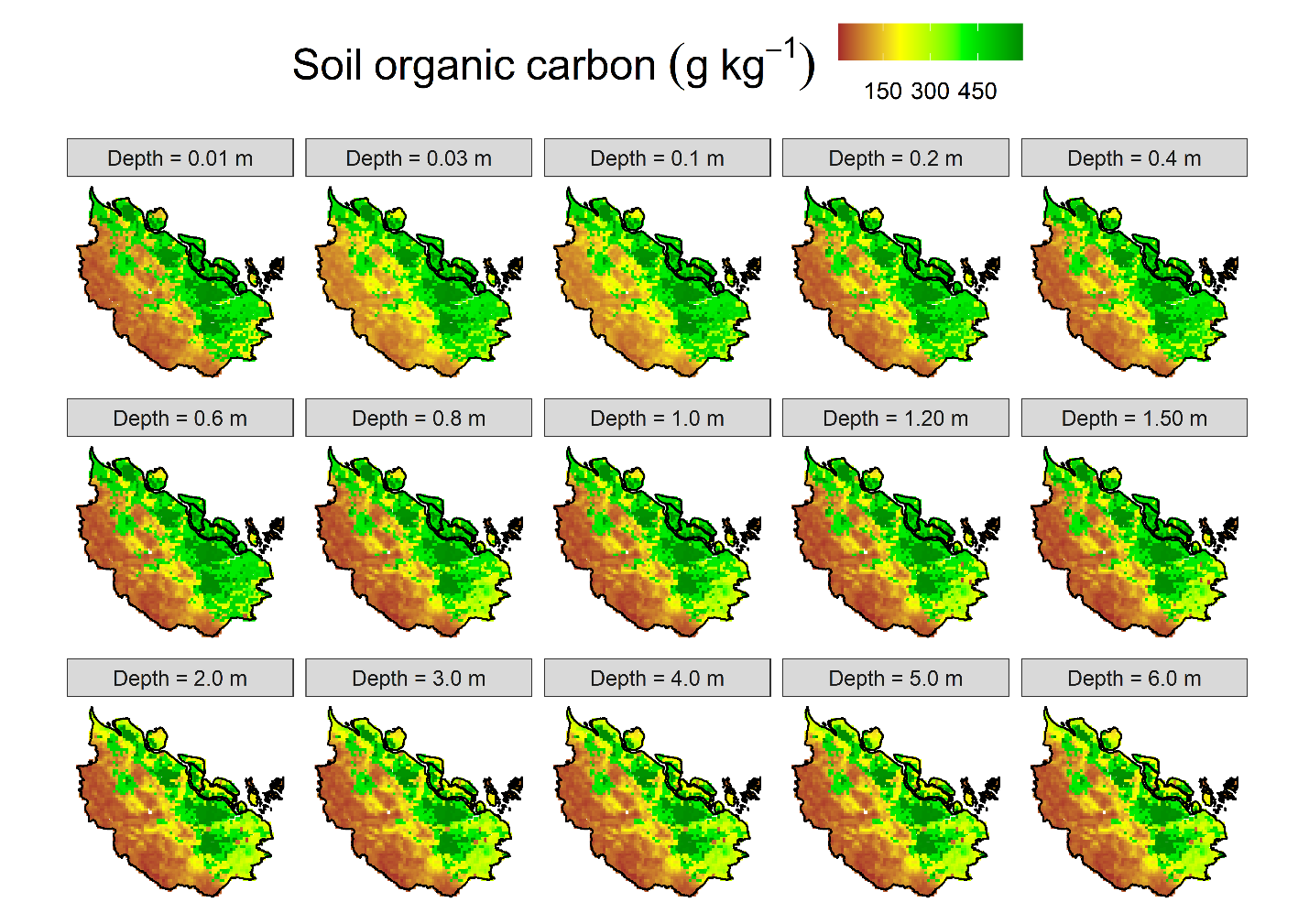


**Supplementary Figure 11. Model inputs of peat or mineral soil organic carbon contents at different depths to simulate hydrologic dynamics across Riau province of Sumatra, Indonesia.** Values for the depths are depths to the bottom of each modelled vertical layer (Supplementary Table 2). The maps were created by using R version 4.0.3 (https://cran.microsoft.com/snapshot/2021-01-17/bin/windows/base/).


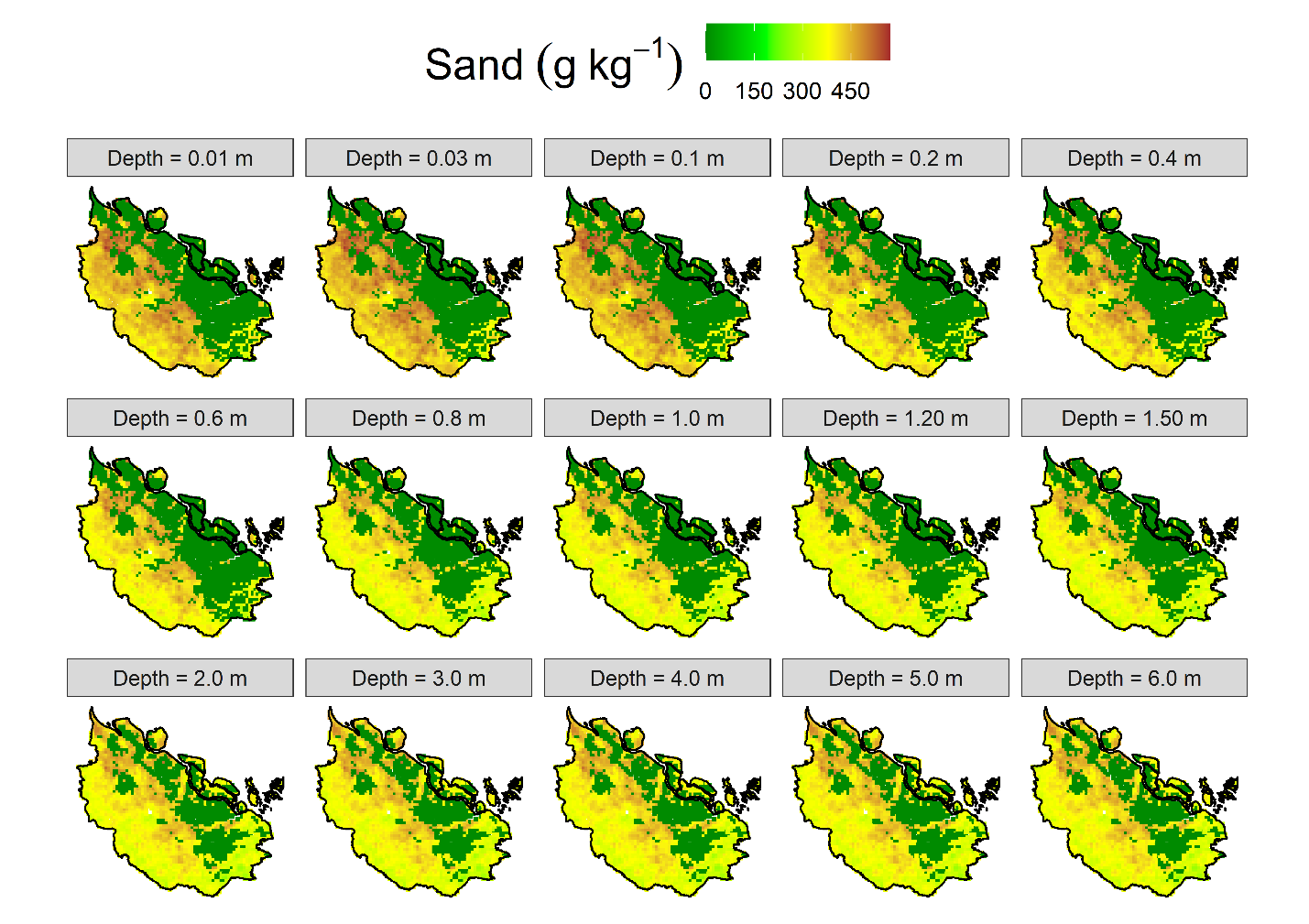


**Supplementary Figure 12. Model inputs of sand contents at different depths to simulate hydrologic dynamics across Riau province of Sumatra, Indonesia.** Values for the depths are depths to the bottom of each modelled vertical layer (Supplementary Table 2). The maps were created by using R version 4.0.3 (https://cran.microsoft.com/snapshot/2021-01-17/bin/windows/base/).
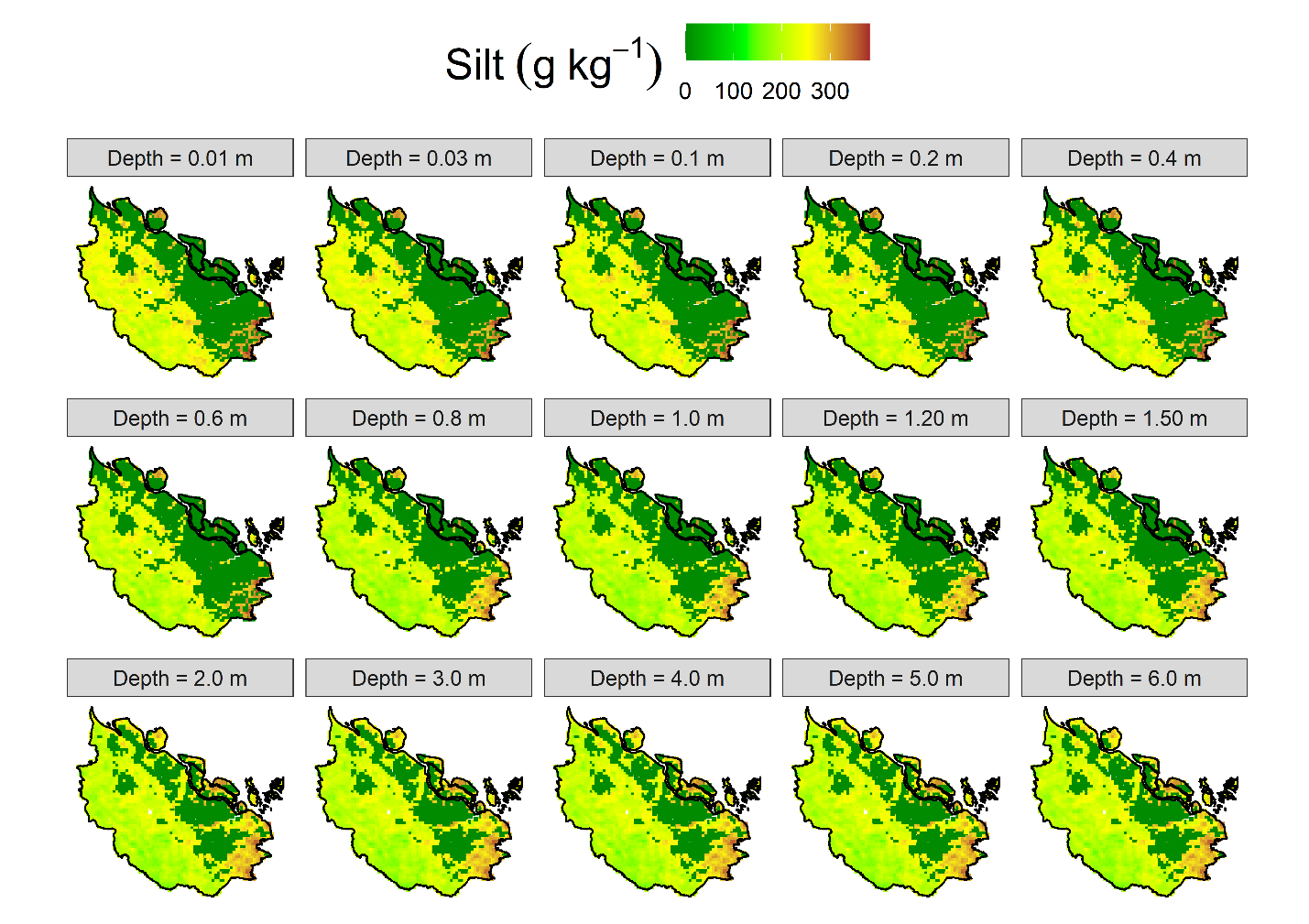


**Supplementary Figure 13. Model inputs of silt contents at different depths to simulate hydrologic dynamics across Riau province of Sumatra, Indonesia.** Values for the depths are depths to the bottom of each modelled vertical layer (Supplementary Table 2). The maps were created by using R version 4.0.3 (https://cran.microsoft.com/snapshot/2021-01-17/bin/windows/base/).


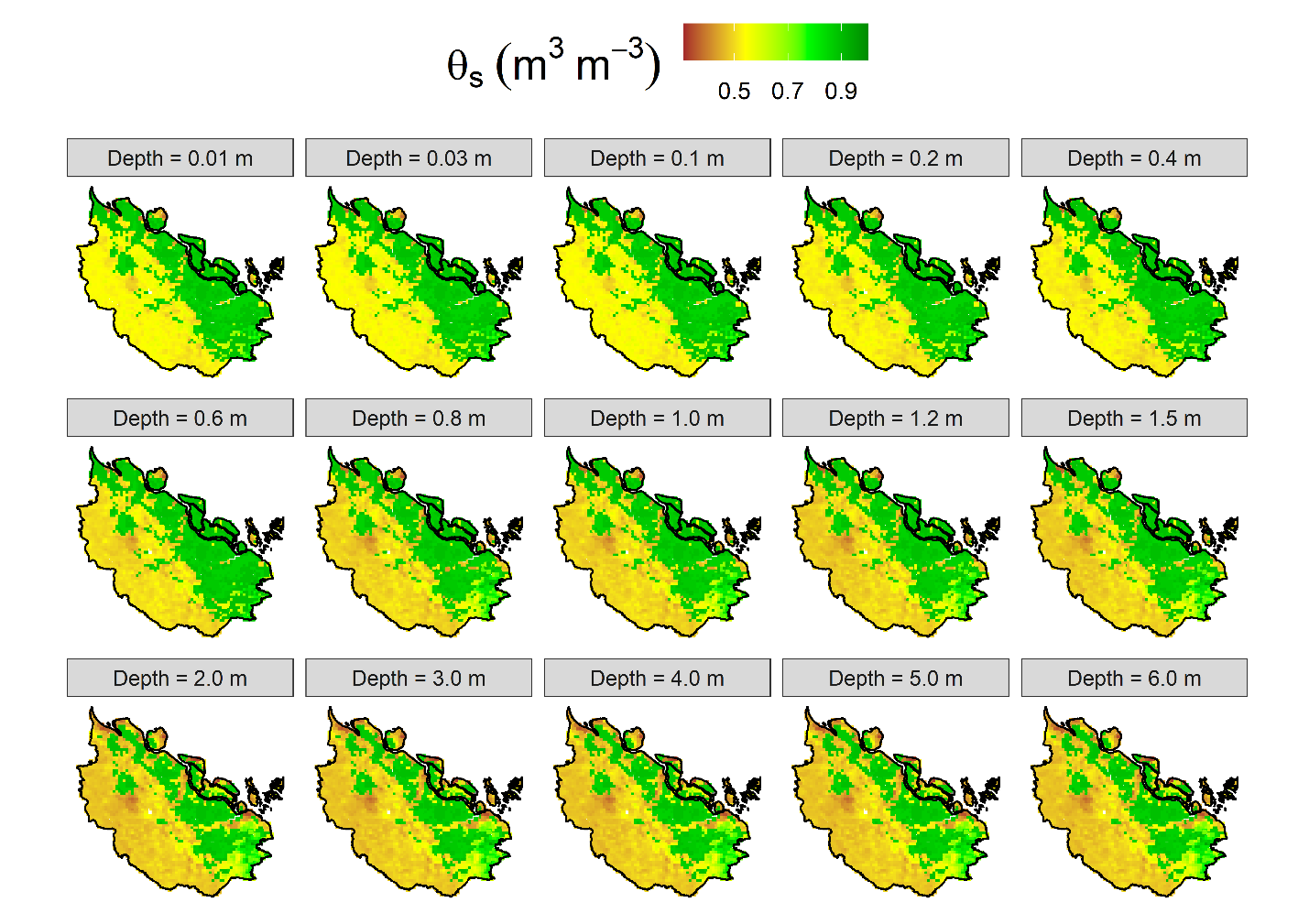


**Supplementary Figure 14. Model inputs of peat or mineral soil water contents at saturation (θ_s_) at different depths to simulate hydrologic dynamics across Riau province of Sumatra, Indonesia.** Values for the depths are depths to the bottom of each modelled vertical layer (Supplementary Equations 20-21). The maps were created by using R version 4.0.3 (https://cran.microsoft.com/snapshot/2021-01-17/bin/windows/base/).


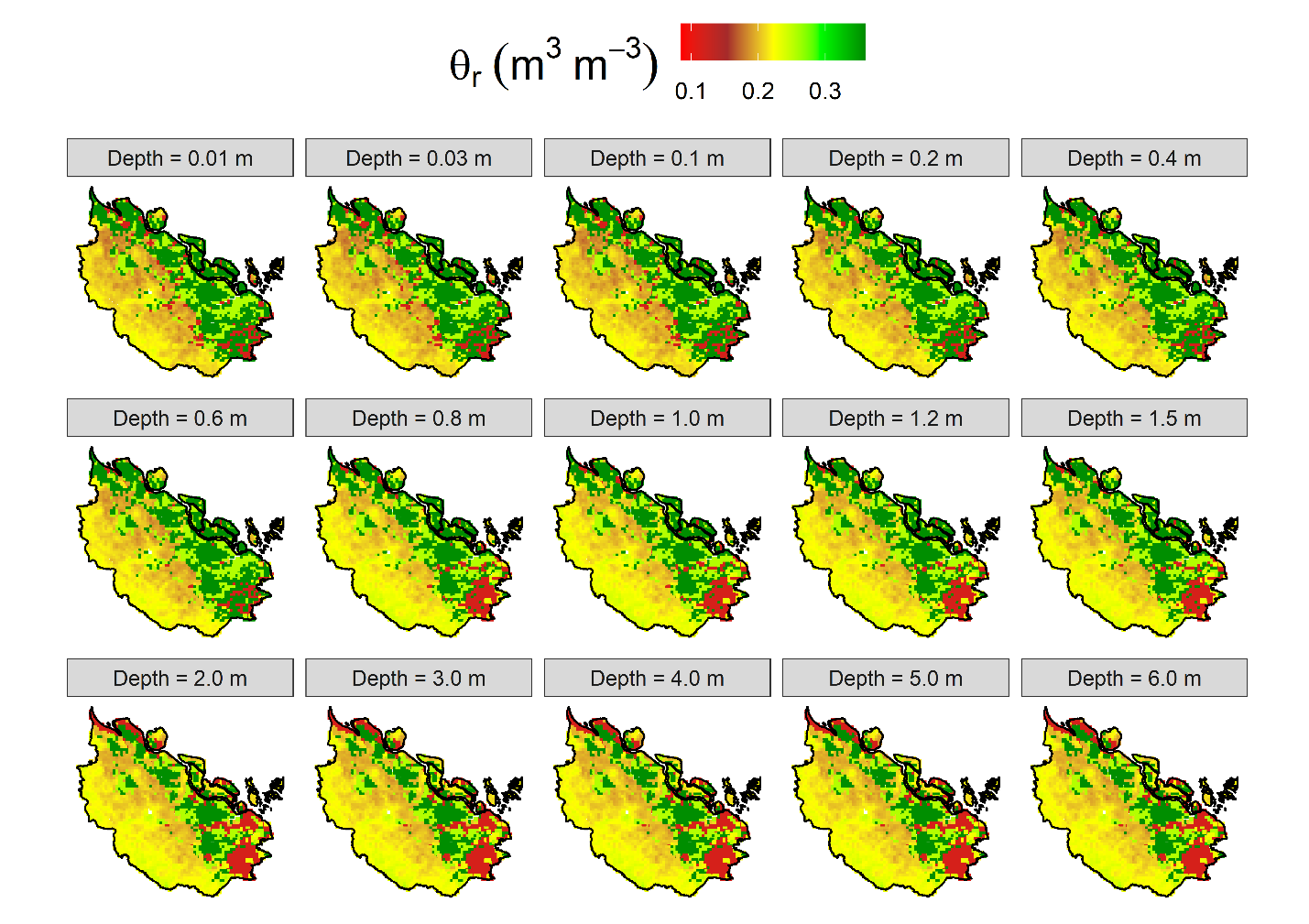


**Supplementary Figure 15. Model inputs of residual peat or mineral soil water contents (θ_r_) at different depths to simulate hydrologic dynamics across Riau province of Sumatra, Indonesia.** θ_r_ represent water contents of a very dry peat or mineral soil (Supplementary Equations 1-3, 12, 15). Values for the depths are depths to the bottom of each modelled vertical layer. The maps were created by using R version 4.0.3 (https://cran.microsoft.com/snapshot/2021-01-17/bin/windows/base/).


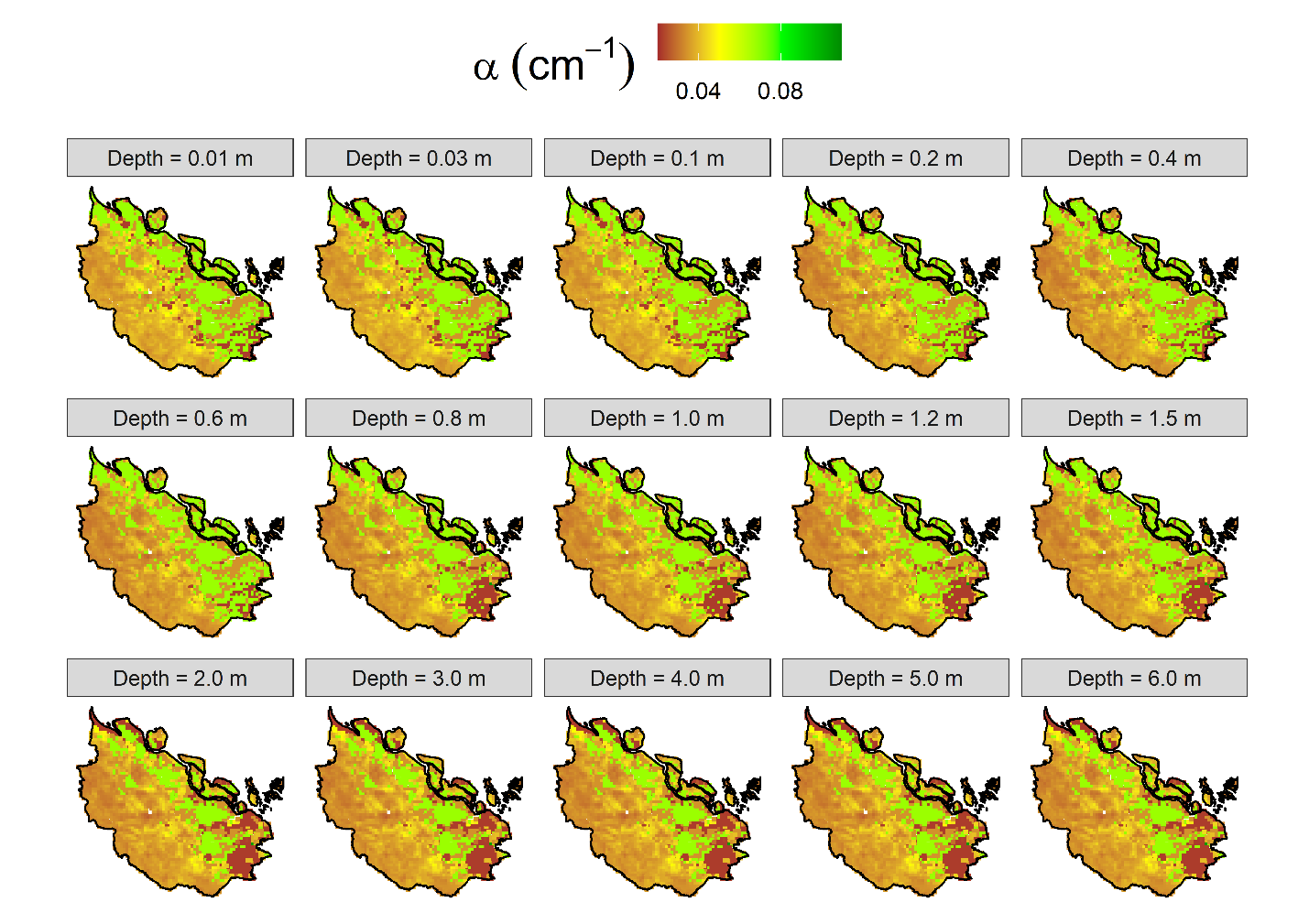


**Supplementary Figure 16. Model inputs of Mualem- van Genuchten peat or mineral soil moisture retention model parameter “α” at different depths to simulate hydrologic dynamics across Riau province of Sumatra, Indonesia**. α determines the inflection point of the sigmoidal peat or mineral soil moisture retention curve (Supplementary Equations 1-3, 14, 17). Values for the depths are depths to the bottom of each modelled vertical layer. The maps were created by using R version 4.0.3 (https://cran.microsoft.com/snapshot/2021-01-17/bin/windows/base/).


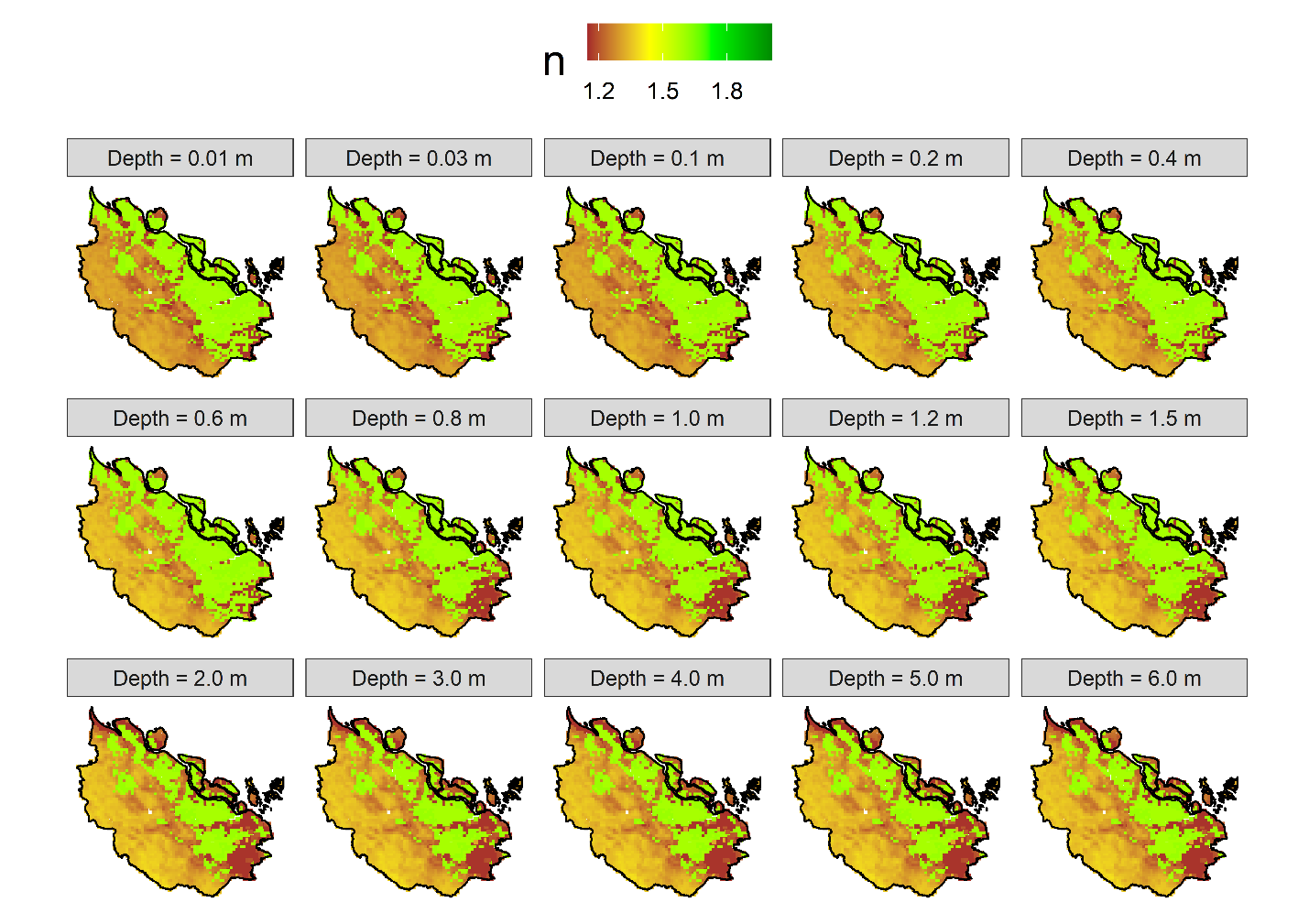


**Supplementary Figure 17. Model inputs of Mualem- van Genuchten peat or mineral soil moisture retention model parameter “*n*” at different depths to simulate hydrologic dynamics across Riau province of Sumatra, Indonesia**. *n* determines the slope of the peat or mineral soil moisture retention curve (Supplementary Equations 1-3, 13, 16). Values for the depths are depths to the bottom of each modelled vertical layer. The maps were created by using R version 4.0.3 (https://cran.microsoft.com/snapshot/2021-01-17/bin/windows/base/).


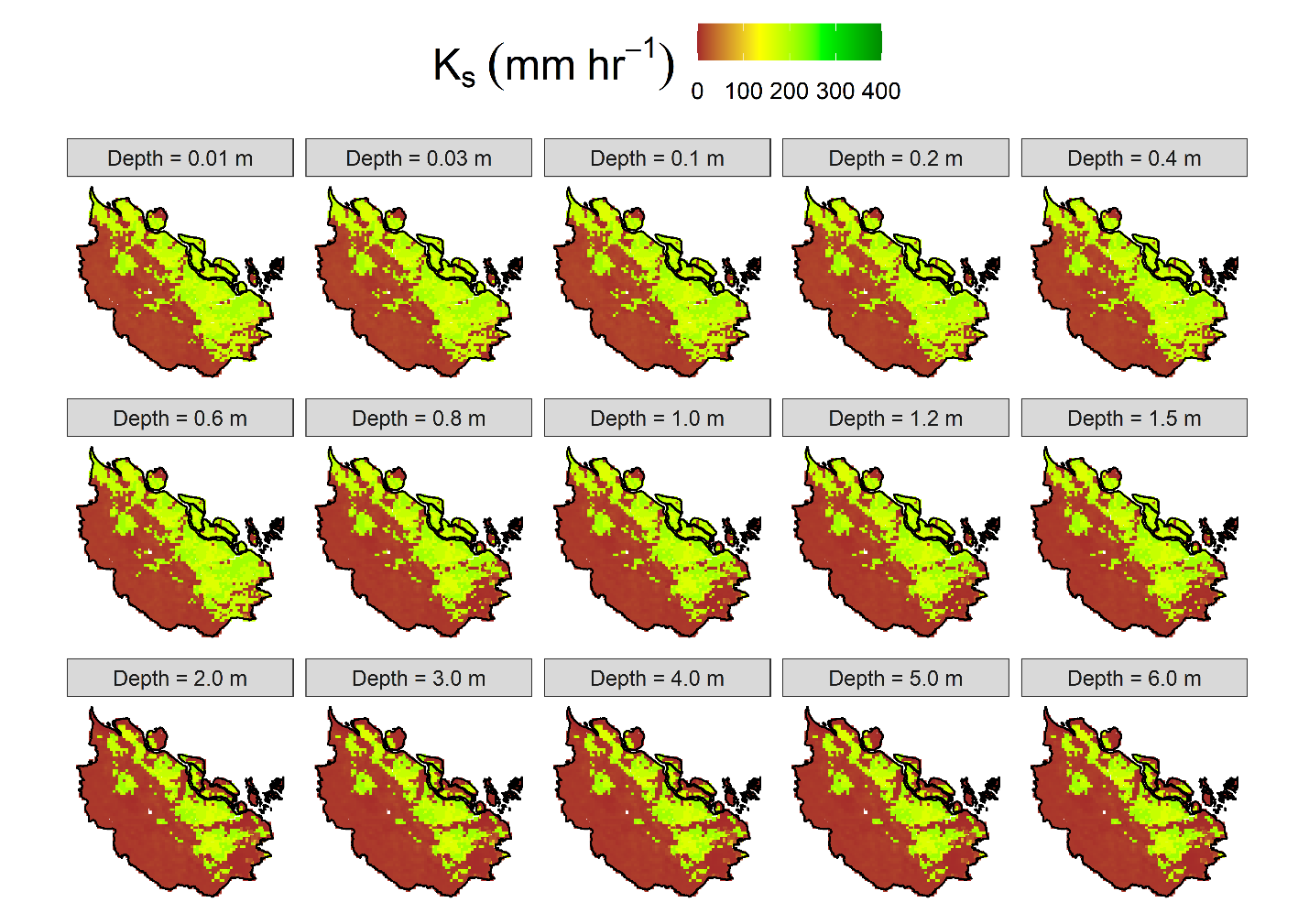


**Supplementary Figure 18. Model inputs of saturated hydraulic conductivity (*K*_s_) at different depths to simulate hydrologic dynamics across Riau province of Sumatra, Indonesia**. Vertical *K*_s_ = lateral *K*_s_ for all layers. Values for the depths are depths to the bottom of each modelled vertical layer (Supplementary Equations 22-23). The maps were created by using R version 4.0.3 (https://cran.microsoft.com/snapshot/2021-01-17/bin/windows/base/).

**Supplementary Table 1. Lateral model boundary conditions** **to simulate hydrologic dynamics across Riau province of Sumatra, Indonesia.** WTDx = external water table depth (WTD) representing average WTD in a watershed adjacent to a grid or average water level of a canal to which the watershed drains. Negative WTDxs are depths below the modelled ground surface^23^

| **Peatland/Non-Peatland** | **Land Use and Land Cover type** | **WTDx (m)** |
| --- | --- | --- |
| Peatland | Small Holder Area/Lowland Mosaic/Lowland Open | -0.7 |
|  | Tall Shrub/Secondary Forest | -0.6 |
|  | Industrial Plantations | -0.8 |
|  | Degraded Peat Swamp Forest | -0.6 |
|  | Mangrove | -0.6 |
|  | Large-Scale Closed Canopy Palm Plantation | -0.8 |
|  | Ferns/Low Shrub | -0.7 |
|  | Cleared/Burnt Area | -0.7 |
|  | Pristine Peat Swamp Forest | -0.6 |
| Non-Peatland | Mangrove | -0.6 |
|  | All other grids | -6.0 |

**Supplementary Table 2. Key model input and validation data and their sources in simulating hydrologic dynamics across Riau province of Sumatra, Indonesia.**

| **Dataset** | **Year** | **Data source** |
| --- | --- | --- |
| Land use and land cover data | 2015 | Land cover distribution in the peatlands dataset^17^for the peatland areas, MODIS (Moderate Resolution Imaging Spectroradiometer) land cover type/dynamics dataset [MCD12C1](http://doi.org/10.5067/MODIS/mcd12c1.006) (<https://modis.gsfc.nasa.gov/data/dataprod/mod12.php>) for the remaining areas. |
| Peat depth |  | <https://www.deltares.nl/en/projects/lidar-data-large-scale-peatland-management-flood-risk-assessment/>  Wetlands International maps of peatland distribution and carbon content^24^. |
| Hourly weather data (shortwave radiation, relative humidity, wind speed, air temperature) | 2008-2015 | ECMWF (European Centre for Medium-Range Weather Forecasts) ERA5 global reanalysis ([https://cds.climate.copernicus.eu](https://cds.climate.copernicus.eu/cdsapp#!/dataset/reanalysis-era5-land?tab=overview))^25^ |
| Daily precipitation (downscaled to 24 hours equally) | 2008-2015 | CHIRPS (Climate Hazards Group InfraRed Precipitation with Station data) (<https://www.chc.ucsb.edu/data>)^26^. |
| Top peat or mineral soil moisture (0-0.05 m depth) | 2015 (April to December) | NASA (National Aeronautics and Space Administration) SMAP (Soil Moisture Active Passive) (<https://smap.jpl.nasa.gov/data/>) |
| Daily active fire count | 2008-2015 | MODIS Active Fire dataset MCD14ML (<https://firms.modaps.eosdis.nasa.gov/active_fire/>)^27.^ |
| Elevation |  | NASA SRTM (Shuttle Radar Topography Mission) Digital Elevation Model dataset  (http://srtm.csi.cgiar.org)^22^.  <https://developers.google.com/earth-engine/datasets/catalog/USGS_SRTMGL1_003> |
| Slope |  | NASA SRTM (Shuttle Radar Topography Mission) Digital Elevation Model dataset  (<http://srtm.csi.cgiar.org>)^22^. (<https://developers.google.com/earth-engine/datasets/catalog/USGS_SRTMGL1_003>) |
| Soil properties at different depths (bulk density, soil organic carbon, sand content, silt content) |  | Global gridded soil information dataset SoilGrids250m (<https://soilgrids.org/>)^28^ |

**Supplementary Table 3. Observed and reported dry bulk density and peat soil organic carbon at different depths for different land use and land cover (LULC) types across different regions of Indonesian peatlands**

| **Region** | **LULC reported in original field study** | **Reclassified modelled LULC (Supplementary Fig. 1)** | **Depth to the bottom of the sampled layer**  **(m)** | **Dry bulk**  **density**  **(Mg m^-3^)** | **Peat soil**  **organic carbon**  **content**  **(g kg^-1^)** |
| --- | --- | --- | --- | --- | --- |
| Central Kalimantan^29^ | undrained forest | pristine peat swamp forest | 0.15 | 0.13 | 555 |
|  |  |  | 0.45 | 0.14 | 573 |
|  |  |  | 0.85 | 0.15 | 615 |
|  | drained forest | degraded peat swamp forest | 0.15 | 0.17 | 555 |
|  |  |  | 0.45 | 0.22 | 638 |
|  |  |  | 0.85 | 0.15 | 593 |
|  |  |  | 1.15 | 0.12 | 604 |
|  | degraded open peatland | ferns/low shrub | 0.15 | 0.2 | 587 |
|  |  |  | 0.45 | 0.12 | 585 |
|  |  |  | 0.85 | 0.14 | 588 |
|  |  |  | 1.15 | 0.15 | 579 |
|  | agricultural open peatland | small holder area/lowland mosaic/lowland open | 0.15 | 0.18 | 586 |
|  |  |  | 0.45 | 0.17 | 583 |
|  |  |  | 0.85 | 0.13 | 596 |
|  |  |  | 1.15 | 0.13 | 582 |
| Central Kalimantan^29^ | rubber plantation | industrial plantations | 0.26 | 0.21 | 512 |
|  |  |  | 1.1 | 0.23 | 542 |
|  |  |  | 2.8 |  | 532 |
|  |  |  | 5.2 |  | 441 |
|  |  |  | 0.23 | 0.23 | 469 |
|  |  |  | 2 | 0.22 | 507 |
|  |  |  | 3.4 |  | 549 |
|  |  |  | 6.5 |  | 313 |
|  |  |  | 0.3 | 0.22 | 497 |
|  |  |  | 0.7 | 0.21 | 485 |
|  |  |  | 1 |  | 531 |
|  |  |  | 6.8 |  | 377 |
|  |  |  | 0.25 | 0.22 | 354 |
|  |  |  | 0.6 | 0.22 | 573 |
|  |  |  | 1.2 |  | 576 |
|  |  |  | 7 |  | 442 |
| South Kalimantan^30^ | agricultural rice paddy | small holder area/lowland mosaic/lowland open | 0.09 | 0.2 | 338 |
|  |  |  | 0.57 | 0.09 | 389 |
|  |  |  | 0.09 | 0.22 | 429 |
|  |  |  | 0.75 | 0.08 | 556 |
|  |  |  | 0.8 |  | 530 |
|  |  |  | 1.35 |  | 404 |
|  |  |  | 1.43 |  | 366 |
|  |  |  | 0.05 | 0.21 | 378 |
|  |  |  | 0.75 | 0.08 | 530 |
|  |  |  | 0.85 |  | 554 |
|  |  |  | 1.7 |  | 423 |
|  |  |  | 1.8 |  | 408 |
|  |  |  | 0.1 | 0.17 | 436 |
|  |  |  | 0.6 | 0.07 | 472 |
|  |  |  | 0.75 |  | 380 |
|  |  |  | 2.2 |  | 443 |
| Central Kalimantan^31^ | rubber plantation | industrial plantations | 0.25 | 0.22 | 354 |
|  |  |  | 0.6 | 0.22 | 573 |
|  |  |  | 1.2 |  | 576 |
|  |  |  | 7 |  | 442 |
|  |  |  | 0.23 | 0.23 | 469 |
|  |  |  | 2 | 0.22 | 507 |
|  |  |  | 3.4 |  | 549 |
|  |  |  | 6.5 |  | 313 |
| South Kalimantan^31^ | agricultural paddy and maize | small holder area/lowland mosaic/lowland open | 0.09 | 0.22 | 429 |
|  |  |  | 0.75 | 0.08 | 556 |
|  |  |  | 0.8 |  | 530 |
|  |  |  | 1.35 |  | 404 |
|  |  |  | 1.43 |  | 366 |
|  |  |  | 0.1 | 0.17 | 436 |
|  |  |  | 0.6 | 0.07 | 472 |
|  |  |  | 0.75 |  | 380 |
|  |  |  | 2.2 |  | 443 |
|  |  |  | 2.3 |  | 251 |
| Riau^31^ | oil palm plantation | industrial plantations | 0.4 | 0.21 | 179 |
|  |  |  | 1.2 | 0.19 | 209 |
|  |  |  | 5.45 |  | 211 |
|  |  |  | 0.4 | 0.22 | 172 |
|  |  |  | 1.1 | 0.23 | 502 |
|  |  |  | 5.8 |  | 495 |
| Jambi^31^ | oil palm plantation | industrial plantations | 0.15 | 0.24 | 491 |
|  |  |  | 0.5 | 0.16 | 240 |
|  |  |  | 1.5 |  | 178 |
|  |  |  | 1.9 |  | 495 |
|  |  |  | 0.35 | 0.17 | 487 |
|  |  |  | 0.6 | 0.17 | 442 |
|  |  |  | 1.6 |  | 260 |
| Central Kalimantan^32^ | undrained forest | pristine peat swamp forest | 0.15 | 0.132 | 553 |
|  |  |  | 1 | 0.141 | 580 |
|  | drained forest | degraded peat swamp forest | 0.15 | 0.17 | 543 |
|  |  |  | 1 | 0.164 | 586 |
|  | drained and burnt forest | cleared/burnt area | 0.15 | 0.199 | 582 |
|  |  |  | 1 | 0.151 | 572 |
|  | agricultural peatland in Kalampangan; drained and cleared for agriculture in 1980s | small holder area/lowland mosaic/lowland open | 0.15 | 0.175 | 579 |
|  |  |  | 1 | 0.152 | 569 |
|  | agricultural peatland in Marang; drained and cleared for agriculture in 1990s | small holder area/lowland mosaic/lowland open | 0.15 | 0.223 |  |
|  |  |  | 1 |  |  |
| Riau^33^ | agricultural land planted with sago palm | small holder area/lowland mosaic/lowland open | 0.2 | 0.13 | 437 |
|  |  |  | 0.4 | 0.11 | 364 |
|  | burnt agricultural land planted with sago palm | cleared/burnt area | 0.2 | 0.17 | 386 |
|  |  |  | 0.4 | 0.14 | 455 |
| Riau^34^ | *Acacia* plantation | industrial plantations | 0.5 | 0.11 |  |
|  |  |  | 0.5 | 0.12 |  |
|  |  |  | 0.5 | 0.08 |  |
|  |  |  | 0.5 | 0.07 |  |
|  |  |  | 0.5 | 0.06 |  |
|  |  |  | 0.5 | 0.06 |  |
| Riau^35^ | Natural forest | pristine peat swamp forest | 0.15 | 0.15 | 500 |
|  | Oil palm plantation (8-10 years old) | industrial plantations | 0.15 | 0.28 | 516 |
|  | Oil palm plantation (3-4 years old) | industrial plantations | 0.15 | 0.31 | 487 |
|  | Rubber plantation (5-10 years old) | industrial plantations | 0.15 | 0.32 | 500 |
|  | Rubber plantation (40-60 years old) | industrial plantations | 0.15 | 0.23 | 479 |
| Riau^36^ | Oil palm plantation | Industrial plantations | 0.15 | 0.28 |  |
|  |  |  | 0.3 | 0.18 |  |
|  |  |  | 0.5 | 0.16 |  |
| West Kalimantan^36^ | Peat swamp forest | pristine peat swamp forest | 0.15 | 0.25 |  |
|  |  |  | 0.3 | 0.18 |  |
|  |  |  | 0.5 | 0.16 |  |

**Supplementary Table 4. Modelled representation of dominant land use and land cover type for each modelled grid in simulating hydrologic dynamics across Riau province of Sumatra, Indonesia.**

| **Land Use and Land Cover type** | **Modelled Plant Functional Type (PFT)** | **Eco-physiological adaptations**  **in modelled PFT** | **Model inputs for plant management** |
| --- | --- | --- | --- |
| Small Holder Area/Lowland Mosaic/Lowland Open | Wetland rice (30% of each grid); dryland rice (30% of each grid); and bushy shrubs (40% of each grid) to represent homestead horticultural plants and other agricultural crops | Dryland rice PFT was identical to the typical field crop PFT ^37^. Wetland rice PFT is the dryland rice PFT modified by increasing the root porosity from 0.05 to 0.33 to represent aquatic adaptation^38^. Bushy shrub PFT is identical to the typical under-storey tropical peat swamp species^1,2^ | Typical planting and harvest dates, and N, P, K fertilizer application rates and timings for wetland and dryland rice^39,40^ |
| Tall Shrub/Secondary Forest | Tropical rainforest tree species^40^ (lower plant density than Lowland Forest) |  | Simulated with self-thinning, herbivory and mortality at rates typical to tropical rainforests^40^ |
| Industrial Plantations | *Acacia* (60% of each grid) and tropical rainforest tree species^40^ to represent rubber (40% of each grid) | *Acacia* is the modification of the tropical rainforest tree speices^41^ by including provisions for biological N_2_ fixation | Simulated with self-thinning, herbivory and mortality at rates typical to tropical rainforests^41^ |
| Degraded Peat Swamp Forest | Tropical peat swamp tree species^1,2^ |  | Simulated with self-thinning, herbivory and mortality at rates typical to tropical rainforests^41^ |
| Mangrove | Tropical peat swamp tree species^1,2^ |  | Simulated with self-thinning, herbivory and mortality at rates typical to tropical rainforests^41^ |
| Large-Scale Closed Canopy Palm Plantation | Tropical peat swamp tree species^1,2^ modified from dicot to monocot | Modified peat swamp tree species PFT by changing from dicot to monocot | Fertilized with N, P, K at typical rates and times^39,42^ |
| Ferns/Low Shrub | Bushy shrub | Identical to the typical under-storey tropical peat swamp species^1,2^ |  |
| Cleared/Burnt Area | Bushy shrub (very low plant density) | Identical to the typical under-storey tropical peat swamp species^1,2^ |  |
| Pristine Peat Swamp Forest | Tropical peat swamp tree species^1,2^ |  | Simulated with self-thinning, herbivory and mortality at rates typical to tropical rainforests^41^ |
| Lowland Forest | Tropical rainforest tree species^41^ |  | Simulated with self-thinning, herbivory and mortality at rates typical to tropical rainforests^41^ |

**Supplementary Table 5. Model inputs of key peat properties at different depths, lateral boundary conditions and dominant plant functional types used in simulating site-level hydrologic dynamics across six tropical peatland sites in Riau province of Sumatra, Indonesia.** WTDx = external water table depth (WTD) representing average WTD in a watershed adjacent to a grid or average water level of a canal to which the watershed drains. Negative WTDxs are depths below the modelled ground surface. SOC = peat or mineral soil organic carbon content^35^, θ_s_ = water content at saturation, θ_r_ = residual water content, *n* = slope parameter of peat or mineral soil moisture retention curve, α = parameter influencing the inflection point of the sigmoidal peat or mineral soil moisture retention curve, K_s_ = saturated hydraulic conductivity. Vertical *K*_s_ = lateral *K*_s_ for all layers. θ_s_, θ_r_, α and *n* are fitted parameters and *K*_s_ were estimated from pedo-transfer function (see Supplementary Methods for details). Peat dry bulk density was measured over the four undrained sites and reported for similar drained sites^35^

|  | | | **Depth (m)**^a^ | | |  |
| --- | --- | --- | --- | --- | --- | --- |
| **Site** | **WTDx (m)** | **Physical and hydrologic**  **properties of peat** | **0 - 0.2** | **0.21 - 0.4** | **0.4 - 6.0** | **Modelled plant**  **functional type (PFT)**  **and plant**  **management** |
| Pristine  peat  swamp | -0.6 | SOC (g kg^-1^)^a^ | 500 | 500 | 500 | Tropical peat swamp tree species^1,2^ simulated with self-thinning, herbivory and mortality at rates typical to tropical rainforests^41^ |
|  |  | Dry bulk density (Mg m^-3^) | 0.16 | 0.12 | 0.14 |  |
|  |  | θ_s_ (m^3^ m^-3^) | 0.88 | 0.91 | 0.89 |  |
|  |  | θ_r_ (m^3^ m^-3^) | 0.36 | 0.32 | 0.36 |  |
|  |  | α (cm^-1^) | 0.068 | 0.106 | 0.068 |  |
|  |  | n (-) | 1.59 | 1.57 | 1.59 |  |
|  |  | K_s_ (mm hr^-1^) | 166.9 | 270.7 | 182.1 |  |
| Zamrud  peat  swamp | -0.6 | SOC (g kg^-1^) ^a^ | 500 | 500 | 500 | Tropical peat swamp tree species^1,2^ simulated with self-thinning, herbivory and mortality at rates typical to tropical rainforests^41^ |
|  |  | Dry bulk density (Mg m^-3^) | 0.13 | 0.11 | 0.14 |  |
|  |  | θ_s_ (m^3^ m^-3^) | 0.9 | 0.91 | 0.89 |  |
|  |  | θ_r_ (m^3^ m^-3^) | 0.32 | 0.32 | 0.36 |  |
|  |  | α (cm^-1^) | 0.106 | 0.106 | 0.068 |  |
|  |  | n (-) | 1.57 | 1.59 | 1.59 |  |
|  |  | K_s_ (mm hr^-1^) | 260.4 | 281.3 | 182.1 |  |
| *Acacia* | -0.6 | SOC (g kg^-1^) ^a^ | 500 | 500 | 500 | Tropical rainforest tree species^41^ modified by including provisions for biological N_2_ fixation; simulated with self-thinning, herbivory and mortality at rates typical to tropical rainforests^41^ |
|  |  | Dry bulk density (Mg m^-3^) | 0.17 | 0.17 | 0.17 |  |
|  |  | θ_s_ (m^3^ m^-3^) | 0.87 | 0.87 | 0.87 |  |
|  |  | θ_r_ (m^3^ m^-3^) | 0.36 | 0.36 | 0.36 |  |
|  |  | α (cm^-1^) | 0.068 | 0.068 | 0.068 |  |
|  |  | n (-) | 1.59 | 1.59 | 1.59 |  |
|  |  | K_s_ (mm hr^-1^) | 159.6 | 159.6 | 159.6 |  |
| Oil palm  and  rubber | -0.6 | SOC (g kg^-1^) ^a^ | 500 | 500 | 500 | Oil palm (occupied 75% of the grid) was tropical peat swamp tree species modified from dicot to monocot. Rubber (occupied 25% of the grid) was identical to the tropical rainforest tree species^41^. The oil palm PFT was fertilized with N, P, K at typical rates and times^39,42^. The rubber PFT was simulated with self-thinning, herbivory and mortality at rates typical to tropical rainforests^41^ |
|  |  | Dry bulk density (Mg m^-3^) | 0.17 | 0.17 | 0.17 |  |
|  |  | θ_s_ (m^3^ m^-3^) | 0.87 | 0.87 | 0.87 |  |
|  |  | θ_r_ (m^3^ m^-3^) | 0.36 | 0.36 | 0.36 |  |
|  |  | α (cm^-1^) | 0.068 | 0.068 | 0.068 |  |
|  |  | n (-) | 1.59 | 1.59 | 1.59 |  |
|  |  | K_s_ (mm hr^-1^) | 159.6 | 159.6 | 159.6 |  |
| *Acacia*/  *Eucalyptus*  (drained) | -1.0 ^b^ | SOC (g kg^-1^) ^a^ | 500 | 500 | 500 | *Acacia* (occupied 60% of the grid) was tropical rainforest tree species^6^ modified by including provisions for biological N_2_ fixation. *Eucalyptus* (occupied 40% of the grid) was identical to the tropical rainforest tree species^41^. Both PFTs were simulated with self-thinning, herbivory and mortality at rates typical to tropical rainforests^41^ |
|  |  | Dry bulk density (Mg m^-3^) | 0.17 | 0.17 | 0.17 |  |
|  |  | θ_s_ (m^3^ m^-3^) | 0.87 | 0.87 | 0.87 |  |
|  |  | θ_r_ (m^3^ m^-3^) | 0.36 | 0.36 | 0.36 |  |
|  |  | α (cm^-1^) | 0.068 | 0.068 | 0.068 |  |
|  |  | n (-) | 1.59 | 1.59 | 1.59 |  |
|  |  | K_s_ (mm hr^-1^) | 159.6 | 159.6 | 159.6 |  |
| Bintangur  peat  swamp  (drained) | -1.0 | SOC (g kg^-1^) ^a^ | 500 | 500 | 500 | Tropical peat swamp tree species^1,2^; simulation of self-thinning, herbivory and mortality at rates typical to tropical rain forests^41^ |
|  |  | Dry bulk density (Mg m^-3^) | 0.16 | 0.12 | 0.14 |  |
|  |  | θ_s_ (m^3^ m^-3^) | 0.88 | 0.91 | 0.89 |  |
|  |  | θ_r_ (m^3^ m^-3^) | 0.36 | 0.32 | 0.36 |  |
|  |  | α (cm^-1^) | 0.068 | 0.106 | 0.068 |  |
|  |  | n (-) | 1.59 | 1.57 | 1.59 |  |
|  |  | K_s_ (mm hr^-1^) | 166.9 | 270.7 | 182.1 |  |

^a^ each modelled peat profile had a total of 15 vertical layers bottomed at 0.01, 0.03, 0.1, 0.2, 0.4, 0.6, 0.8, 1.0, 1.2, 1.5, 2.0, 3.0, 4.0, 5.0, and 6.0 m. Each of the 15 layers had the properties of the corresponding depth ranges

^b^reset at -0.8m for a brief period (between June and December) during the simulation year (2010) to represent site WTD management (see Supplementary Methods for details)

**Supplementary references**

[1] Mezbahuddin, M., Grant, R. F. & Hirano, T. How hydrology determines seasonal and interannual variations in water table depth, surface energy exchange, and water stress in a tropical peatland: Modeling versus measurements. *J. Geophys. Res. Biogeosci.* **120**, 2132-2157 (2015).

[2] Mezbahuddin, M., Grant, R. F. & Hirano, T. Modelling effects of seasonal variation in water table depth on net ecosystem CO_2_ exchange of a tropical peatland. *Biogeosciences* **11**, 577-599 (2014).

[3] Grant, R. F., Desai, A. R. & Sulman, B. N. Modelling contrasting responses of wetland productivity to changes in water table depth. *Biogeosciences* **9**, 4215-4231 (2012).

[4] Mezbahuddin, M., Grant, R. F. & Flanagan, L. B. Modeling hydrological controls on variations in peat water content, water table depth, and surface energy exchange of a boreal western Canadian fen peatland. *J. Geophys. Res. Biogeosci.* **121**, 2216-2242 (2016).

[5] Dimitrov, D. D., Grant, R. F., Lafleur, P. M. & Humphreys, E. R. Modeling the effects of hydrology on gross primary productivity and net ecosystem productivity at Mer Bleue bog. *J. Geophys. Res. Biogeosci.* **116**, G04010 (2011).

[6] Dimitrov, D. D., Bhatti, J. S. & Grant, R. F. The transition zones (ecotone) between boreal forests and peatlands: Modelling water table along a transition zone between upland black spruce forest and poor forested fen in central Saskatchewan. *Ecol. Modell.* **274**, 57-70 (2014).

[7] Van Genuchten, M. T. A closed‐form Supplementary Equation for predicting the hydraulic conductivity of unsaturated soils. *Soil Sci. Soc. Am. J.* **44**, 892-898 (1980).

[8] Ippisch, O., Vogel, H. J. & Bastian, P. Validity limits for the van Genuchten–Mualem model and implications for parameter estimation and numerical simulation. *Adv. Water Resour.* **29**, 1780-1789 (2006).

[9] Hodnett, M. G. & Tomasella, J. Marked differences between van Genuchten soil water-retention parameters for temperate and tropical soils: a new water-retention pedo-transfer functions developed for tropical soils. *Geoderma* **108**, 155-180 (2002).

[10] Kozan, O. *Catastrophe and Regeneration in Indonesia’s Peatlands: Ecology, Economy and Society* Ch. 10 (National University of Singapore Press, 2016).

[11] Taufik, M., Veldhuizen, A. A., Wösten, J. H. M. & van Lanen, H. A. J. Exploration of the importance of physical properties of Indonesian peatlands to assess critical groundwater table depths, associated drought and fire hazard. *Geoderma* **347**, 160-169 (2019).

[12] Kurnain, A., Notohadikusumo, T., Radjagukguk, B. and Hastuti, S. Peat soil properties related to degree of decomposition under different land use systems. *Int. Peat J.* **11**, 67-77 (2001).

[13] Kurnain, A. Hydrophysical properties of ombrotrophic peat under drained peatlands. *Int. Agrophys.* **33***,* 277-283 (2019).

[14] Saxton, K. E. & Rawls, W. J. Soil water characteristic estimates by texture and organic matter for hydrologic solutions. *Soil Sci. Soc. Am. J.* **70**, 1569-1578 (2006).

[15] Willmott, C. J. On the validation of models. *Phys. Geogr.* **2**, 184-194 (1981).

[16] Willmott, C. J. Some comments on the evaluation of model performance. *Bull. Amer. Meteor. Soc.* **63**, 1309-1313 (1982).

[17] Miettinen, J., Shi, C. & Liew, S. C. Land cover distribution in the peatlands of Peninsular Malaysia, Sumatra and Borneo in 2015 with changes since 1990. *Glob. Ecol. Conserv.* **6**, 67-78 (2016).

[18] Hirano, T., *et al*. Effects of disturbances on the carbon balance of tropical peat swamp forests. *Glob. Chang. Biol.* **18**, 3410-3422 (2012).

[19] Beckwith, C. W., Baird, A. J. & Heathwaite, A. L. Anisotropy and depth related heterogeneity of hydraulic conductivity in a bog peat: I. Laboratory measurements. *Hydrol. Process* **17**, 89-101 (2003).

[20] Liu, H., Janssen, M. & Lennartz, B. Changes in flow and transport patterns in fen peat as a result of soil degradation. *Eur. J. Soil Sci.* **67**, 763-772 (2016).

[21] Taufik, M., Setiawan, B. I. & Van Lanen, H. A. Increased fire hazard in human-modified wetlands in Southeast Asia. *Ambio*, **48**, 363-373 (2019).

[22] Jarvis, A., Reuter, H. I., Nelson, A., & Guevara, E. Hole-filled seamless SRTM data V4. <http://srtm.csi.cgiar.org> (2008).

[23] Evans, C. D., *et al*. Rates and spatial variability of peat subsidence in *Acacia* plantation and forest landscapes in Sumatra, Indonesia. *Geoderma* **338**, 410-421 (2019).

[24] Wahyunto, R. S. & Suparto, S. H. Maps of area of peatland distribution and carbon content in Sumatra and Kalimantan. <https://data.globalforestwatch.org/datasets/indonesia-peat-lands> (2004).

[25] Hersbach, H., *et al*. The ERA5 global reanalysis*, Q. J. Roy. Meteor. Soc.* **146**, 1999–2049 (2020).

[26] Funk, C., Peterson, P., Landsfeld, M., Pedreros, D., Verdin, J., Shukla, S., Husak, G., Rowland, J., Harrison, L., Hoell, A. & Michaelsen, J. The climate hazards infrared precipitation with stations-a new environmental record for monitoring extremes. *Sci. Data* **2**, 1-21 (2015).

[27] Giglio, L., Schroeder, W. & Justice, C. O. The collection 6 MODIS active fire detection algorithm and fire products. *Remote Sens. Environ.* **178**, 31-41 (2016).

[28] Hengl, T., *et al.* SoilGrids250m: Global gridded soil information based on machine learning. *PLoS One* **12**, e0169748 (2017).

[29] Könönen, M., Jauhiainen, J., Laiho, R., Kusin, K. and Vasander, H. Physical and chemical properties of tropical peat under stabilised land uses. *Mires Peat* **16**, 1-13 (2015).

[30] Nugroho, K. & Sarwani, M. Characterizing the cultivated lowland peat soils in two physiography positions in Kalimantan, Indonesia. *Int. Res. J. Agric. Sci. Soil Sci.* **3**, 246-255 (2013).

[31] Hikmatullah, H. & Sukarman, S. Physical and Chemical PRoperties of Cultivated Peat Soils in Four TRial Sites of ICCTF in Kalimantan and Sumatra, Indonesia. *J. Tropic. Soils* **19**, 131-141 (2014).

[32] Jauhiainen, J., *et al.* Nitrous oxide fluxes from tropical peat with different disturbance history and management. *Biogeosciences* **9**, 1337-1350 (2012).

[33] Iskandar, I, Irawan A. F. & Suwarno, M. Chemical, physical and microbial characteristics of soil cultivated with sago palm in Tebing Tinggi Island, Riau Islands during and one year after land and forest fire. Preprint <https://www.researchgate.net/publication/316188248_Chemical_Physical_and_Microbial_Characteristics_of_Peat_Soil_Cultivated_with_Sago_Palm_in_Tebing_Tinggi_Island_Riau_Islands_During_and_One_Year_after_Land_and_Forest_Fire> (2016).

[34] Jauhiainen, J., Hooijer, A. & Page, S. E. Carbon dioxide emissions from an Acacia plantation on peatland in Sumatra, Indonesia. *Biogeosciences***9**, 617-630 (2012).

[35] Nurulita, Y., *et al*. The assessment of the impact of oil palm and rubber plantations on the biotic and abiotic properties of tropical peat swamp soil in Indonesia. *Int. J. Agric. Sustain.* **13**, 150-166 (2015).

[36] Marwanto, S., Watanabe, T., Iskandar, W., Sabiham, S. & Funakawa, S. Effects of seasonal rainfall and water table movement on the soil solution composition of tropical peatland. *Soil Sci. Plant Nutr.* **64**, 386-395 (2018).

[37] Mezbahuddin, S., *et al*. Assessing effects of agronomic nitrogen management on crop nitrogen use and nitrogen losses in the Western Canadian prairies. *Front. Sustain. Food Syst.* **4**, 512292 (2020).

[38] Cronk, J. K. & Fennessy, M. S. *Wetland plants: biology and ecology* (CRC press, 2016).

[39] Food and Agriculture Organization of the United Nations. *Fertilizer use by crop in Indonesia*. Preprint <https://www.fao.org/3/y7063e/y7063e07.htm> (2005).

[40] Ehara, H., Suwignyo, R. A. & Sakagami, J. I. Swamp rice cultivation in South Sumatra, Indonesia. *Trop. Agric. Dev.* **59**, 35-39 (2015).

[41] Grant, R. F., *et al.* Modeling the carbon balance of Amazonian rain forests: resolving ecological controls on net ecosystem productivity. *Ecol. Monogr.* **79**, 445-463 (2009).

[42] Woittiez, L. S., Slingerland, M., Rafik, R. & Giller, K. E. Nutritional imbalance in smallholder oil palm plantations in Indonesia. *Nutr. Cycl. Agroecosys.* **111**, 73-86 (2018).
